# LRH‐1/NR5A2 targets mitochondrial dynamics to reprogram type 1 diabetes macrophages and dendritic cells into an immune tolerance phenotype

**DOI:** 10.1002/ctm2.70134

**Published:** 2024-12-19

**Authors:** Nadia Cobo‐Vuilleumier, Silvia Rodríguez‐Fernandez, Livia López‐Noriega, Petra I. Lorenzo, Jaime M. Franco, Christian C. Lachaud, Eugenia Martin Vazquez, Raquel Araujo Legido, Akaitz Dorronsoro, Raul López‐Férnandez‐Sobrino, Beatriz Fernández‐Santos, Carmen Espejo Serrano, Daniel Salas‐Lloret, Nila van Overbeek, Mireia Ramos‐Rodriguez, Carmen Mateo‐Rodríguez, Lucia Hidalgo, Sandra Marin‐Canas, Rita Nano, Ana I. Arroba, Antonio Campos Caro, Alfred CO Vertegaal, Alejandro Martín‐Montalvo, Franz Martín, Manuel Aguilar‐Diosdado, Lorenzo Piemonti, Lorenzo Pasquali, Roman González Prieto, Maria Isabel García Sánchez, Decio L. Eizirik, Maria Asuncion Martínez‐Brocca, Marta Vives‐Pi, Benoit R. Gauthier

**Affiliations:** ^1^ Andalusian Center of Molecular Biology and Regenerative Medicine‐CABIMER Junta de Andalucía‐University of Pablo de Olavide‐University of Seville‐CSIC Seville Spain; ^2^ Nadia Cobo‐Vuilleumier and Petra I Lorenzo Centro de Investigación Biomédica en Red de Diabetes y Enfermedades Metabólicas Asociadas (CIBERDEM) Madrid Spain; ^3^ Immunology Department Germans Trias i Pujol Research Institute Autonomous University of Barcelona Badalona Spain; ^4^ ULB Center for Diabetes Research Medical Faculty Université Libre de Bruxelles (ULB) Brussels Belgium; ^5^ Cell and Chemical Biology Leiden University Medical Centre Leiden The Netherlands; ^6^ Pompeu Fabra University Barcelona Spain; ^7^ Department of Endocrinology and Nutrition University Hospital Virgen Macarena Sevilla Spain; ^8^ Diabetes Research Institute IRCCS Ospedale San Raffaele Milan Italy; ^9^ Department of Endocrinology and Nutrition University Hospital Puerta del Mar, Institute of Research and Innovation in Biomedical Sciences of Cádiz (INiBICA). University of Cádiz (UCA) Cádiz Spain; ^10^ Department of Cell Biology Faculty of Biology University of Sevilla Sevilla Spain; ^11^ Biobank of the Andalusian Public Health System Node Hospital Virgen Macarena Sevilla Spain

**Keywords:** autoimmune diseases, drug development, immune tolerance, pancreatic islets

## Abstract

**Background:**

The complex aetiology of type 1 diabetes (T1D), characterised by a detrimental cross‐talk between the immune system and insulin‐producing beta cells, has hindered the development of effective disease‐modifying therapies. The discovery that the pharmacological activation of LRH‐1/NR5A2 can reverse hyperglycaemia in mouse models of T1D by attenuating the autoimmune attack coupled to beta cell survival/regeneration prompted us to investigate whether immune tolerisation could be translated to individuals with T1D by LRH‐1/NR5A2 activation and improve islet survival.

**Methods:**

Peripheral blood mononuclear cells (PBMCs) were isolated from individuals with and without T1D and derived into various immune cells, including macrophages and dendritic cells. Cell subpopulations were then treated or not with BL001, a pharmacological agonist of LRH‐1/NR5A2, and processed for: (1) Cell surface marker profiling, (2) cytokine secretome profiling, (3) autologous T‐cell proliferation, (4) RNAseq and (5) proteomic analysis. BL001‐target gene expression levels were confirmed by quantitative PCR. Mitochondrial function was evaluated through the measurement of oxygen consumption rate using a Seahorse XF analyser. Co‐cultures of PBMCs and iPSCs‐derived islet organoids were performed to assess the impact of BL001 on beta cell viability.

**Results:**

LRH‐1/NR5A2 activation induced a genetic and immunometabolic reprogramming of T1D immune cells, marked by reduced pro‐inflammatory markers and cytokine secretion, along with enhanced mitohormesis in pro‐inflammatory M1 macrophages and mitochondrial turnover in mature dendritic cells. These changes induced a shift from a pro‐inflammatory to an anti‐inflammatory/tolerogenic state, resulting in the inhibition of CD4^+^ and CD8^+^ T‐cell proliferation. BL001 treatment also increased CD4^+^/CD25^+^/FoxP3^+^ regulatory T‐cells and Th2 cells within PBMCs while decreasing CD8+ T‐cell proliferation. Additionally, BL001 alleviated PBMC‐induced apoptosis and maintained insulin expression in human iPSC‐derived islet organoids.

**Conclusion:**

These findings demonstrate the potential of LRH‐1/NR5A2 activation to modulate immune responses and support beta cell viability in T1D, suggesting a new therapeutic approach.

**Key Points:**

- LRH‐1/NR5A2 activation in inflammatory cells of individuals with type 1 diabetes (T1D) reduces pro‐inflammatory cell surface markers and cytokine release.
- LRH‐1/NR5A2 promotes a mitohormesis‐induced immuno‐resistant phenotype to pro‐inflammatory macrophages.
- Mature dendritic cells acquire a tolerogenic phenotype via LRH‐1/NR5A2‐stimulated mitochondria turnover.
- LRH‐1/NR5A2 agonistic activation expands a CD4^+^/CD25^+^/FoxP3^+^ T‐cell subpopulation.
- Pharmacological activation of LRH‐1/NR5A2 improves the survival iPSC‐islets‐like organoids co‐cultured with PBMCs from individuals with T1D.

## Graphical abstract

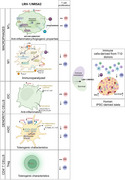

## 1 INTRODUCTION

Type 1 diabetes mellitus (T1D) is one of the most prevalent chronic paediatric illnesses affecting 1.75 million individuals under the age of 20 years (https://diabetesatlas.org/atlas/t1d‐index‐2022/). A recent study estimated that in 2021, there were 355 900 new cases of T1D in children and adolescents worldwide. This number is projected to increase to 476 700 by 2050.^1^ T1D is considered a T‐cell‐mediated autoimmune disease caused by a disruption in the balance between T‐regulatory cells (Tregs) and T‐effector cells (Teffs; CD4^+^ and CD8^+^ cytotoxic T‐cells) that respond to islet‐associated self‐antigens.^2^ This breakdown in immune homeostasis or ‘tolerance’ leads to β‐cell destruction, resulting in insulin (INS) deficiency, hyperglycaemia and the lifelong necessity for INS supplementation in afflicted individuals.^3^ Several immunosuppressive therapies have been developed based on this immune dysfunction origin, and their beneficial effects—at least in part—have been demonstrated in clinical trials. One of these, teplizumab (anti‐CD3 derivative), delayed the development of T1D in individuals ‘at‐risk’ by 2 years and it was recently approved by the Food and Drug Administration. While marking a significant breakthrough, the ability of the drug to delay the disease by only 2 years underscores the underlying complexity of the disease.^2^ Considering the latter, the contribution of β‐cells in the pathogenesis of T1D has gained momentum, as evidenced by the expression of nearly 80% of the T1D susceptible gene variants in the β‐cells.^4^, ^5^ These gene variants modulate pro‐inflammatory signals and cause vulnerability to endoplasmic reticulum and oxidative stress, triggering cell dysfunction and apoptosis. Due to the highly vascularised islet microenvironment, β‐cell stress signals reach circulating immune cells, initiating a cross‐talk. This cross‐talk may ultimately destroy β‐cells and is further influenced by other genetic and environmental factors.^6^, ^7^, ^8^ Therefore, new effective disease‐modifying therapies for T1D should aim to resolve this detrimental dialogue by simultaneously targeting both immune and islet cells.^9^

Nuclear receptors (NRs) play pivotal roles in a wide range of physiological and pathological processes.^10^ They regulate metabolic pathways that control cellular energy balance, survival and environmental adaptability. NRs are also crucial for whole‐organism functions such as development, metabolism, reproduction, immune response and tissue regeneration. The fact that NRs activities can be controlled by ligands has made them attractive targets for drug development, with potential therapeutic applications.^11^, ^12^ One such NR is the liver receptor homolog 1 (LRH‐1, *a.k.a*. NR5A2), which has emerged as a promising drug target for diseases like diabetes, pancreatic cancer, non‐alcoholic fatty liver disease and metabolic syndrome.^13^, ^14^, ^15^, ^16^, ^17^, ^18^ Our previous work provided an early proof‐of‐concept that the specific pharmacological activation of LRH‐1/NR5A2 using a small chemical agonist (BL001) could therapeutically impede the progression of hyperglycaemia in two mouse models of T1D (NOD and RIP‐B7.1) without long‐term adverse effects, validating the benefits of targeting this NR.^19^ BL001 coordinated in vivo the resolution, rather than the suppression, of the autoimmune attack by increasing the number of anti‐inflammatory M2 while decreasing the number of pro‐inflammatory M1 macrophages and concomitantly increasing the number of tolerogenic dendritic cells (DCs) and Tregs. In parallel, BL001 stimulated β‐cell regeneration via trans‐differentiation and improved cell survival, the latter involving the PTGS2/PGE_2_/PTGER1 signalling cascade.^19^, ^20^, ^21^

Given the strong disease‐modifying properties of LRH‐1/NR5A2 activation in mouse models of T1D and aiming to evaluate the potential for clinical applicability of this strategy, herein we expanded our studies to human primary immune cells obtained from individuals with T1D as well as from healthy donors. LRH‐1/NR5A2 expression has been described in macrophages, dendritic and T‐cells, arguing for a direct and specific impact of BL001 on these immune cells.^22^, ^23^, ^24^ In T‐cells, LRH1/NR5A2 regulates various functions including maturation and proliferation, which directly impacts immune responses and could be pivotal in modulating autoimmune diseases like T1D.^24^ Our endpoint was to define the molecular mode of action of LRH‐1/NR5A2 agonistic activation in T1D cells, which is especially relevant considering that the diabetic milieu impedes the anti‐inflammatory characteristics of both human macrophages and DCs.^25^, ^26^, ^27^

## 2 MATERIALS AND METHODS

### 2.1 Sex as a biological variable

Both male and female participants were recruited for this study. No gender differences were observed, so data from both sexes were combined for all experiments.

### 2.2 Subjects

Samples of 30–50 mL peripheral blood were collected by venipuncture from individuals with T1D (73) and control healthy subjects (37) in BD Vacutainer Sodium Heparin tubes (BD Biosciences, San Jose, CA, USA) (Table 1). Inclusion criteria were 20–55 years of age, body mass index (BMI) 18–30 kg/m^2^ and disease evolution longer than 5 years. Exclusion criteria were being under immunosuppressive or anti‐inflammatory treatment or undergoing pregnancy or breastfeeding. Donors were informed of the procedure and signed a written consent before blood extraction. The collection and processing of personal and clinical data from included subjects were limited to those data necessary to achieve the objectives of the study, and the data collected were processed with adequate precautions to ensure confidentiality and compliance with applicable data privacy protection laws and regulations.

**TABLE 1:** Characteristics and clinical data of blood donors. Samples were collected from three independent national hospitals. N/A, not applicable.

	Healthy subjects	Individuals with T1D
*n* =	37	73
Gender (F/M)	20F/17 M	33F/40 M
Age (years)	33 ± 9	37 ± 10
BMI (kg/m^2^)	23 ± 3	24 ± 4
Age at diagnosis (years)	N/A	20 ± 12
Progression (years)	N/A	16 ± 11
HbA1c (%)	N/A	7 ± 0.9
Insulin dose (IU/kg/day)	N/A	0.53 ± 0.17

### 2.3 Peripheral blood mononuclear cell and monocyte isolation

Peripheral blood mononuclear cells (PBMCs) were isolated using Ficoll Paque or Histopaque 1077 density gradient centrifugation. The PBMC layer was extracted and washed with PBS. Cells were resuspended in PBS, 2% FBS and 1 mM EDTA buffer, and monocytes were then isolated using the magnetic EasySep Human CD14^+^ Selection kit (STEMCELL Technologies, Vancouver, BC, Canada) following the manufacturer's instructions. When the purity of CD14 marker in the selected fraction was greater than 90%, monocytes were cultured in 24‐well plates (Labclinics, Barcelona, Spain) at a concentration of 10^6^ cells/mL in either X‐VIVO 15 media (Lonza, Basel, Switzerland) or RPMI 1640 media (ThermoFisher Scientific), both supplemented with 2% male AB human serum (Biowest Nuaillé, France), 100 IU/mL penicillin (Sigma–Aldrich and Normon SA, Madrid, Spain), 100 µg/mL streptomycin (Sigma–Aldrich, Madrid, Spain). The negatively selected fraction of PBMCs was cryopreserved in FBS (ThermoFisher Scientific) with 10% dimethylsulfoxide (Sigma–Aldrich, Saint Louis, MO, USA) at a 10–20 × 10^6^ cells/mL and stored for later use.

### 2.4 Monocyte‐derived macrophages and DCs

Purified CD14^+^ monocytes were derived into either monocyte‐derived macrophages (MDMs) or dendritic cells (DCs). For MDMs, cells were treated with 1000 IU/mL rhGM‐CSF (Prospec, Rehovot, Israel) for 3 days to generate naïve/primed M1_0_ and subsequently with a cocktail containing 20 ng/mL of INFγ (Immunotools, Friesoythe, Germany) and 10 ng/mL of LPS (Sigma–Aldrich) to promote the pro‐inflammatory M1 phenotype. Alternatively, monocytes were treated with 1000 IU/mL rhIL‐4 and 1000 IU/mL rhGM‐CSF (Prospec) for 6 days to obtain DCs. Media and cytokine stimuli were replenished on day 4. DCs were either cultured with 20 µg/mL human INS (Sigma–Aldrich) to obtain immature DCs (iDCs) or adding a cytokine cocktail (CC) consisting of tumour necrosis factor (TNF)α (1000 IU/mL; Immunotools), IL1β (2000 IU/mL; Immunotools) and prostaglandin E2 (PGE2, 1 µM, Cayman Chemical, Ann Arbor, MI, USA) to obtain mature DCs (mDCs). All derived cell types were cultivated at 1 × 10^6^ cells/mL density and maintained at 37°C in 5% CO^2^.

### 2.5 CD4^+^ T‐cell isolation

The CD4^+^ T‐cell subpopulation was isolated from the CD14^−^ a fraction of PBMCs using the Easysep human CD4^+^ T‐cell isolation kit (Stemcell Technologies) per the manufacturer's instructions. CD4^+^ T‐cells were cultured in RPMI 1640 media (ThermoFisher Scientific), supplemented with 2% male AB human serum (Biowest Nuaillé, France), 100 IU/mL penicillin (Sigma–Aldrich and Normon SA), 100 µg/mL streptomycin (Sigma–Aldrich). Subsequently, CD4^+^ T‐cells were activated using the TransAct reagent (Milteny) for 24 h before a single dose of 10 µM BL001 was added to the culture media.

### 2.6 Culture of the induced pluripotent stem cell line and differentiation

The induced pluripotent stem cell (iPSC) line 1023A, which originated from bone marrow, displayed a normal karyotype (XY), typical stem cell colony morphology and expressed pluripotency markers as described by Lytrivi et al.^28^ These iPSCs were cultured in Essential E8 medium (ThermoFisher Scientific, ES) on plates coated with Matrigel. To differentiate the iPSCs into pancreatic beta‐like cells, a 7‐step protocol was employed as previously outlined.^28^, ^29^, ^30^, ^31^, ^32^ Following differentiation, stage 7 cell aggregates were dispersed using a phosphate‐buffered saline solution containing 0.5 mM EDTA at room temperature for 6 min, followed by an 8‐min treatment with Accumax (Sigma–Aldrich) and gentle pipetting to detach the cells. To stop the dissociation process, knockout serum (ThermoFisher Scientific) was added, and then the cells were pelleted, resuspended in a medium supplemented with 10 µmol/L ROCK inhibitor and 50 µM 3‐isobutyl‐1‐methylxanthine (Sigma) for overnight culture and seeded on Matrigel™‐coated plates prepared in DMEM‐F12 at a density of 5 × 10^4^ cells per 6.4 mm well.

### 2.7 Co‐culture of PBMCs with iPSC‐derived islet‐like organoids

PBMCs from T1D donors were activated using T Cell TransAct at 1:100 in RPMI‐1640 with 2% human serum and penicillin–streptomycin for 24 h at 37°C. Human iPSC (hiPSC)‐derived islet‐like organoids were dispersed using Accumax for 8 min and seeded on Matrigel‐coated 24‐well plates at 50 000 cells per well in medium containing Ham's F‐10, 0.75% fatty acid‐free BSA, Glutamax and penicillin–streptomycin. After 24 h of recovery, activated PBMCs were added to the dispersed cells at a 10:1 ratio in Ham's F‐10 medium supplemented with 2% human serum, 0.75% BSA, Glutamax and penicillin–streptomycin. Co‐cultures were treated with 10 µM BL001 at 24, 48 h and 30 min prior to the termination of the experiment. Media were collected at 48 h, PBMCs were analysed by flow cytometry, and iPSC‐derived islet‐like cells were processed for RNA extraction, TUNEL assay and immunofluorescence.

### 2.8 BL001 treatment

Three consecutive doses of 10 µM BL001 (stock solution of 10 mM in 100% DMSO) were added to the culture media of PBMCs, M1_0_, M1, iDCs and mDCs and the co‐culture of hiPSCs‐derived islet‐like organoids with PBMCs at 0, 24 h and 30 min prior to cell analysis or processing. Administering a final dose 30 min prior to the termination of the experiment ensured that BL001 was active during the time of analysis, allowing us to capture both its immediate and cumulative effects on cellular responses.

### 2.9 Flow cytometry

Subpopulations of immune cells were characterised by flow cytometry (FACSCalibur and FACSAria I; BD Biosciences, Madrid, Spain), using either Zombie Violet 421 (BioLegend) or 7aad (BD Biosciences) for viability assessment and the following antibodies for (1) MDMs phenotyping: CD14 APC/FITC, CD80 PE, CD86 APC, CD163 PE‐Vio615/BV421, CD200R PE, CD206 FITC/BV711 and CD209 FITC and (2) DCs phenotyping: CD11c APC, CD25 PE, CD86 FITC, HLA class I FITC, HLA class II FITC, CD14 PE and CD40 APC, CD36 APCCy7, TIM4 APC, αvβ5 integrin PE, CD54 PECy7, CXCR4 APCCy7, CCR2 APC, DC‐SIGN‐APC, PD‐L1 PECy7 and CCR7 PECy7 (Table 2). Regulatory T cells were evaluated using the REAfinity™ Treg Phenotyping Kit (Table 2). T helper cell phenotyping was performed using CD4 Viogreen, CD183 PE, CD194 APC, CD196 BV423. Data were analysed using the FlowJo V9 (Tree Star) or the FCS Express software (De Novo Software, Pasadena, USA).

**TABLE 2:** Reagents and resources used in this study.

Reagent or resource	Supplier	Identifier
Antibodies		
CCR7 PECy7	BD Biosciences	Cat#557648
Annexin V PE	Immunotools	Cat#31490014 7
CD3 PE	Immunotools	Cat#21620034
CD4 APC	Immunotools	Cat#21278046
CD8 FITC	Immunotools	Cat#21810083
CD11c APC	Immunotools	Cat#21487116
CD14 PE	Immunotools	Cat#21620144
CD25 PE	Immunotools	Cat#21810254
CD40 APC	Immunotools	Cat#21270406
CD86 FITC	Immunotools	Cat#21480863
HLA class I FITC	Immunotools	Cat#21159033
HLA class II FITC	Immunotools	Cat#21388993
αvβ5 integrin PE	BioLegend	Cat#920007
CD36 APCCy7	BioLegend	Cat#336213
CD54 PECy7	BioLegend	Cat#353115
CCR2 APC	BioLegend	Cat#357207
CXCR4 APCCy7	BioLegend	Cat#306528
TIM4 APC	BioLegend	Cat#354008
DC‐SIGN APC	BioLegend	Cat#330108
PD‐L1 PECy7	BioLegend	Cat#329717
CTV Cell Proliferation Kit	ThermoFisher Scientific	Cat#C34557
CSFE	ThermoFisher Scientific	Cat#C34554
Zombie Violet 421	BioLegend	Cat#423114
CD4	Miltenyi Biotec	Cat#130‐122‐994
CD8	eBioscience	Cat#45‐0088‐41
CD14	BioLegend	Cat#325604
CD25	Miltenyi Biotec	Cat#130‐122‐994
CD80	Miltenyi Biotec	Cat#130‐123‐253
CD86	BioLegend	Cat#305412
CD163	Miltenyi Biotec	Cat#130‐112‐126
CD183	Miltenyi Biotec	Cat#130‐120‐452
CD194	Miltenyi Biotec	Cat#130‐117‐376
CD196	Miltenyi Biotec	Cat#130‐127‐189
CD206	Miltenyi Biotec	Cat#130‐095‐131
CD200R	Miltenyi Biotec	Cat#130‐111‐290
CD209	Miltenyi Biotec	Cat#130‐092‐873
FoxP3	Miltenyi Biotec	Cat#130‐122‐994
ACTIN	Sigma‐Aldrich	Cat#A5441
ATF4	Cell Signaling	Cat#11815
INSULIN	Sigma‐Aldrich	Cat#I2018
GLUCAGON	Cell Signaling	Cat#2760
Chemicals, peptides and recombinant proteins		
BL001	In house	In house
rhIL‐4	Prospec	Cat#CYT‐211
rhGM‐CSF	Prospec	Cat#CYT‐221
Human insulin	Sigma‐Aldrich	Cat#I3536
TNFα	Immunotools	Cat#11343015
IL‐1β	Immunotools	Cat#11340013
PGE2	Cayman Chemical	Cat#14010
Streptozotocin	Sigma‐Aldrich/Merck	Cat#S0130‐1G
Penicillin	Sigma‐Aldrich/Merck	Cat#P0781
Streptomycin	Sigma‐Aldrich/Merck	Cat#P0781
INFγ	Immunotools	Cat#11343536
LPS	Sigma‐Aldrich/Merck	Cat# L6529
Critical commercial assays		
Cell Mito Stress Test Kit	Agilent	Cat#103015‐100
Treg Phenotyping Kit, anti‐human, REAfinity	Miltenyi Biotec	Cat#130‐122‐994
Human Cytokine Array C5	RayBiotech	Cat#AAH‐CYT‐5‐2
Acetyl Coenzyme A assay kit	Sigma‐Aldrich	Car#MAK039‐1KT
CD4+ T cell isolation kit	Stemcell Technology	Cat#17952
Click‐iT Plus TUNEL assay	Invitrogen	Cat#C10619
EpiQuik Total Histone Extraction Kit	Epigentek	Cat#OP‐0006‐100
EpiQuik Histone H3 Modification Multiplex Assay Kit	Epigentek	Cat#P‐3100‐96
Deposited data		
RNAseq dataset	This paper	
Proteomic dataset	This paper	
Oligonucleotides		
Primer: ATF4 Fw: TCAAACCTCATGGGTTCTCC Rv: GTGTCATCCAACGTGGTCAG		
Primer: CCNI Fw: GCACAGATGGATAGCTCC Rv: CTTTGTCACAGGTCACCA		
Primer: FoxP3 Fw: GGCACAATGTCTCCTCCAGAGA Rv: CAGATGAAGCCTTGGTCAGTGC		
Primer: GDF15 Fw: ACCTGCACCTGCGTATCTCT Rv:CGGACGAAGATTCTGCCAG		
Primer: IFNγ Fw: GAGTGTGGAGACCATCAAGGAAG Rv: TGCTTTGCGTTGGACATTCAAGTC		
Primer: IL1B Fw: AGCTACGAATCTCCGACCAC Rv: CGTTATCCCATGTGTCGAAGAA		
Primer: INS Fw: AGGCTTCTTCTACACACCCAAG Rv: CACAATGCCACGCTTCTG		
Primer: NLRP3 Fw: CGTGAGTCCCATTAAGATGGAGT Rv: CCCGACAGTGGATATAGAACAGA		
Primer: NR5A2/LRH1 Fw: GCACAGGAGTTAGTGGCAAA Rv: TTCCTGGACACCTTCTACCA		
Primer: RSP9 Fw: AAGGCCGCCCGGGAACTGCTGAC Rv: ACCACCTGCTTGCGGACCCTGATA		
Software and algorithms		
FlowJo	Tree Star Inc	www.flowjo.com
FCS Express	De Novo Software	https://denovosoftware.com/
Fiji	Imagej	https://imagej.net/software/fiji/downloads
Prism	GraphPad	https://www.graphpad.com/
Adobe Photoshop	Adobe	https://www.adobe.com/es/
ImageJ	Imagej	https://imagej.nih.gov/ij/
Cytoscape	Cytoscape	https://cytoscape.org/index.html
SRplot	SRplot	https://www.bioinformatics.com.cn/srplot
Other		
BD Vacutainer Sodium Heparin tubes	BD	Cat#366667
Ficoll Paque PLUS	Cytiva	Cat#17144003
Histopaque 1077	Sigma‐Aldrich	Cat#10771
EasySep Human CD14 Positive Selection kit II	STEMCELL Technologies	Cat#17858
EasySep Human CD4+ T cell isolation kit	STEMCELL Technologies	Cat#17952
Accutase	ThermoFisher Scientific	Cat#00‐4555‐56
7‐AAD	BD Biosciences	Cat#559925
MitoTracker Green	ThermoFisher Scientific	Cat#M7514
MitoTracker Red	ThermoFisher Scientific	Cat#M7512
T‐Cell TransAct human	Miltenyi Biotec	Cat#130‐111‐160
On‐target plus Human NR5A2 (2495) siRNA‐smart pool	Dharmacon	L‐003430‐00‐0005
On‐target plus non‐targeting control pool	Dharmacon	D‐001810‐10‐20

### 2.10 Cytokine profiling

Media were collected from MDMs and DCs, treated or not with BL001, and analysed using the RayBiotech Human Cytokine Array C5, which interrogates 80 cytokines simultaneously (RayBiotech, Norcross, USA). Membranes were scanned and analysed using the ImageJ/Fiji software. Results were then normalised using the internal controls of the membranes, and the relative signal intensity are represented. To determine the cytokines released in the medium derived from the co‐culture of hiPSC‐derived islet‐like organoids with PBMCs, the medium was collected, centrifuged and the supernatant was frozen until further analysis. Then, the BD™ Cytometric Bead Array (CBA) Human Th1/Th2 Cytokine Kit II (Catalog No. 551809) was used, following the manufacturer's protocol recommendations.

### 2.11 Autologous T cell proliferation assays

Immune cells cultured in each condition were co‐cultured with autologous T lymphocytes to determine their capacity to modulate T cell proliferation. Briefly, PBMCs from the same donor were thawed to be stained with the CellTrace Violet (CTV) or CFSE Cell Proliferation kit (ThermoFisher Scientific) following the manufacturer's instructions. After staining, cells were suspended in either RPMI 1640 or X‐VIVO 15 complete media at a final concentration of 10^6^ cells/mL, and 100 000 cells were plated in 96‐well round bottom plates (Labclinics). T cell activation was induced using a cocktail of CD3 and CD28 antibodies (T Cell TransAct; Miltenyi Biotech). Stained and activated PBMCs were co‐cultured with either MDMs, DCs or CD4^+^ T‐cells subject to the various experimental conditions at a 10:1 or 2:1 ratio (10^5^ PBMCs:10^4^ DCs; 2 × 10^4^ PBMCs: 10^4^ MDMs; 2 × 10^4^ PBMCs: 10^4^ CD4^+^ T‐cells) in triplicates. After up to 6 days of co‐culture in the incubator at 37°C and 5% CO_2_, cells were washed with 150 µL PBS per well at 400×*g* for 5 min and incubated for 20 min at 4°C with a staining mix containing CD3 PE, CD4 APC/Viogreen and CD8 FITC/PerCP‐Cy5.5 staining and 7‐AAD/Zombie Violet 421. Regulatory T cells were evaluated using the REAfinity™ Treg Phenotyping Kit. Cells were then washed in PBS and T‐cell proliferation was analysed by flow cytometry. Data were analysed using the FlowJo software (Tree Star Inc.).

### 2.12 Transcriptome profiling

Total RNA was isolated from human primary immune cells using the RNeasy Plus Micro Kit (Qiagen). RNA integrity number (RIN) values were evaluated using Bioanalyzer® 2100 (pico assay) and their profiles were accepted for preparing libraries for NGS (RIN > 8.40). RNA‐seq libraries were performed by the Genomic Facility at CABIMER, using the kit Illumina Stranded TOTAL RNA preparation RIBO‐ZERO PLUS and sequenced on a NovaSeq 6000 platform with an average of 30 million reads per sample. Reads were mapped and quantify using Salmon (version 1.5.0) with default parameters to the human transcriptome (assembly GRCh38) downloaded from GENCODE genome with default parameters. Differential expression was analysed using Deseq2 using a paired sample design. Gene set enrichment analysis (GSEA) was performed using clusterProfiler (version 4.0).

### 2.13 Proteomic profiling

MDMs and DCs were lysed in 8 M urea/10 mM HEPES (pH 8.0). Reduction/alkylation was applied with dithiothreitol and chloroacetamide. Proteins were first digested with 500 ng of rLys‐C (Promega) for 5 h, diluted below 2 M urea with 50 mM ammonium bicarbonate and digested overnight with 500 ng of trypsin (Promega). Resulting peptides were desalted with C18 StageTips^33^ and analysed by liquid chromatography with tandem mass spectrometry. Macrophage samples were analysed in a Q‐Exactive Orbitrap mass spectrometer (Thermo Scientific, Germany) with a Top10 data‐dependent acquisition mode basically as in Ref. ^34^. Mass spectrometry RAW files was analysed using MaxQuant (v2.1.3.0).^35^ Search was performed against the Homo Sapiens UniProt reference proteome (29 August 2022) according to standard settings with the following modifications: three missed cleavages allowed in digestion maximum number of modifications 3. Match‐between‐runs and Label‐Free Quantification (LFQ) were enabled, not allowing Fast LFQ. For the DC data, samples were analysed in a timsTOF SCP (Bruker Daltonics) with a data independent acquisition parallel accumulation serial fragmentation (dia‐PASEF) mode as previously reported.^36^ Next, DIA‐PASEF data were analysed using DIA‐NN (version 1.8.1).^37^, ^38^ Using an in silico predicted spectral library using the Homo sapiens UniProt reference proteome for including only canonical proteins (19 January 2024) enabling one mis cleavage with trypsin and 100–1700 *m/z* range. Output from MaxQuant and DIA‐NN were processed for statistical analysis in Perseus .^39^ Values were log2 transformed, and potential contaminants and reverse peptides were removed. Samples were grouped in experimental categories and proteins not identified in every replicate in at least one condition were removed. Missing values were imputed with default variables and results exported into MS Excel, and paired *t*‐tests were performed. Volcano plots were constructed for data visualisation using the VolcaNoseR web^40^ (https://huygens.science.uva.nl/VolcaNoseR2/). We explored the protein–protein interaction (PPI) network among differentially expressed proteins (DEPs) in MDMs using the online search tool for the retrieval of interacting genes/proteins (STRING v11) that comprise a database of known and predicted PPIs.^41^ Networks were then visualised in the Cytoscape software (version 3.9.1), using the yfile circular layout algorithm.^42^ Alternatively, the SRplot webserver platform was used to interrogate GO datasets for DEPs in DCs.^43^

### 2.14 Acetyl coenzyme A measurement

M1, treated or not with BL001, were washed in PBS and then lysed in 1 M perchloric acid. The lysate was centrifuged at 10 000×*g* for 10 min at 4°C. The deproteinised supernatant was neutralised with potassium bicarbonate and the neutral pH confirmed with Whatman® indicator papers. The acetyl CoA content was then determined using the acetyl coenzyme A assay kit as per the instructions of the manufacturer (Sigma–Aldrich). An acetyl‐CoA standard curve was plotted in the range of 0–100 pmol. Fluorescence was measured using a Varioskan Flash spectrophotometer. For normalisation by protein content, the protein pellets were solubilised first in 0.2 N NaOH and then solubilised in a buffer containing 7 M urea, 2 M thiourea, Tris 50 mM pH 8.8, as previously described.^44^ The protein content was determined using the Quick Start Bradford Protein assay (Bio‐Rad). Acetyl CoA content was then normalised to protein content.

### 2.15 Mitochondrial bioenergetic and fitness

Mitochondrial bioenergetics of MDMs and DCs were measured using the Seahorse XF Cell Mito Stress Test Kit and the XF24 Extracellular Flux Analyzer (Agilent), as previously described.^45^ Briefly, after overnight culture, cells were washed and replenished with Seahorse assay media (Seahorse Bioscience), supplemented with 1 mM pyruvate and 2 mM glutamine. Where applicable, 10 µM BL001 and 1 mM palmitate conjugated to 0.17 mM BSA (150 mM NaCl, pH 7.2) were added to designated wells. Plates were incubated in a CO_2_‐free incubator at 37°C for 1 h to allow temperature and pH equilibration, after which the oxygen consumption rate (OCR) and extracellular acidification rate (ECAR) were measured in the XF24 Extracellular Flux Analyzer over a period of 95 min. Mitochondrial processes were examined through sequential injections of oligomycin (4 µM) at min 21, carbonyl cyanide 4‐(trifluoromethoxy) phenylhydrazone (FCCP; 2 µM) at min 45, 5 µM antimycin A/Rotenone at min 78. At the end of the measurement, cells in each well were counted using the Scepter™ 2.0 Cell Counter to normalise the data. Only the OCR and ECAR for the basal condition was considered, for which the areas under the curves are presented in pertinent figures. Mitochondrial fitness, as assessed by the number of non‐functional mitochondria in mDCs treated or not with BL001, was determined by flow cytometry using the probes MitoTracker Green that binds covalently to mitochondrial proteins, thus providing an assessment of mass and MitoTracker Red that is taken up by polarised mitochondria thus gauging function. Non‐functional mitochondria were then determined by the ratio of MitoTracker Green^High^ over MitoTracker Red^low^.^46^ Fluorescent images were acquired using a Leica TCS SP5 confocal microscope.

### 2.16 siRNA silencing

On‐target plus NR5A2 siRNA‐smart pool or control on‐target plus non‐targeting pool were used for silencing studies in PBMCs (Table 2) as previously described.^19^ RNA was extracted 48 h post‐transfection.

### 2.17 RNA extraction and quantitative real‐time PCR

Total RNA from was extracted using the RNeasy Micro Kit (Qiagen, Madrid, SP). Complementary DNA using 0.1 to 1 µg RNA was synthesised using the Superscript III Reverse Transcriptase (Invitrogen‐Thermo Fisher Scientific, Madrid, Spain). The qRT‐PCR was performed on individual cDNAs using SYBR green (Roche).^19^ Gene‐specific primers were selected using a human housekeeping gene database (HRT Atlas v1.0 database)^47^ and the sequences are listed in Table 2. Expression levels were normalised to various reference genes, including CCNI, ACTIN, CYCLOPHILIN and RSP9. The relative gene expression was calculated using the standard curve‐based method.^48^

### 2.18 Protein analysis

MDMs were disrupted in a RIPA lysis buffer containing protease (P8340; Merck/Sigma‐Aldrich) and phosphatase inhibitors (P0044, P5726; Merck/Sigma–Aldrich). Western blots were performed according to standard methods.^45^ Antibodies employed are provided in Table 2. For the acetylation studies, histones were extracted from MDMs using the EpiQuik Total Histone Extraction Kit (Epigentek, NY, USA). Acetylation levels of H3 were then evaluated with the EpiQuik Histone H3 Modification Multiplex Assay Kit, following the manufacturer's protocol (Epigentek).

### 2.19 Immunofluorescence analyses

Dispersed human hiPSC‐derived islet‐like organoids cultured on Matrigel™ (Corning/ThermoFisher Scientific)‐coated coverslips were fixed with 4% paraformaldehyde for 10 min, permeabilised with 0.2% Triton X‐100 in PBS for 10 min and blocked with 2% BSA in PBS for 1 h. After blocking, the cells were subjected to the Click‐iT™ Plus TUNEL Assay Kits for in situ apoptosis detection or to specific primary antibodies for INS and glucagon (GCG). Primary antibodies (Table 2) were incubated overnight at 4°C. Following the primary antibody incubation, the cells were washed with PBS and incubated with the respective secondary antibodies (Table 2) for 1 h at room temperature. After another PBS wash, the nuclei were counterstained with 0.0001% 4′,6‐diamidino‐2‐phenylindole (DAPI; Sigma–Aldrich), and coverslips were mounted using a fluorescent mounting medium (DAKO). Epifluorescence microscopy images were acquired with a Leica DM6000B microscope. Quantification of fluorescence signals was performed using Fiji software.

### 2.20 Statistical analysis

**FIGURE 1:**
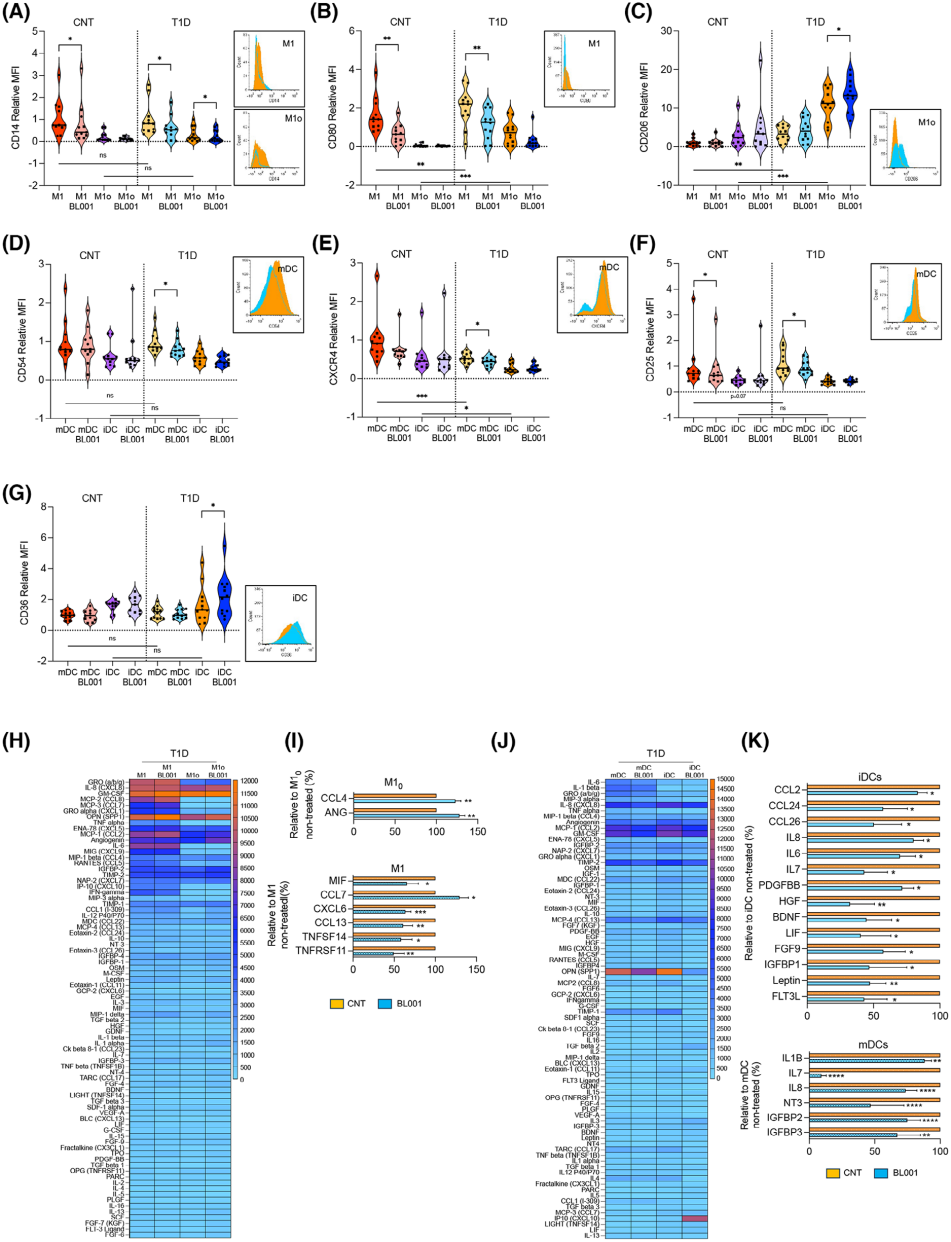
LRH‐1/NR5A2 activation reduces the pro‐inflammatory immune cell phenotype and cytokine secretion in T1D. Monocytes from healthy and T1D donors were differentiated into monocytes‐derived macrophages (MDM) of resting or pro‐inflammatory (M1_0_ or M1) type and immature or mature dendritic cells (iDCs or mDCs). LRH1/NR5A1 activation was achieved by administering 10 µM BL001 every 24 h for 48 h, with a final dose 30 min before analysis. Macrophages cell surface markers (A) CD14, (B) CD80, (C) CD206 and DC cell surface markers, (D) CD54, (E) CXCR4, (F) CD25 and (G) CD36 were analysed by flow cytometry. Measurements were normalised to the mean fluorescence intensity (MFI) of M1 or mDC from controls. Data are presented as means ± SEM from *n* = 10 healthy and T1D individuals for MDM and DC markers. Male, black squares and female, black circles. Statistical analysis was performed as described in the materials and methods section: **p < 0.01 and ***p < 0.001. Flow cytometry histograms (untreated: orange and BL001 treated: blue) are shown only for the markers with statistically significant differences in T1D samples. Heatmaps depicting the average cytokine levels from individual donors for (H) MDM and (J) DCs. Bar graphs show relative changes in cytokine secretion for (I) MDM and (K) DCs, with treated values compared with their untreated counterpart. Only significantly altered cytokines are shown. Data are presented as percent changes compared with DMSO (non‐treated) for each cytokine. Unpaired Student *t*‐test *p < 0.05, **p < 0.01, ***p < 0.001, ****p < 0.001 compared with DMSO (non‐treated).

Statistical analyses were performed using GraphPad Prism software version 10 (GraphPad Software, La Jolla, USA). Data are presented as the mean ± SEM. More specifically for Figure 1, normality of the data was assessed using the Shapiro–Wilk test. When the data deviated from normality (*p* < 0.05), the Friedman test was used to compare conditions within patient and control groups. Significant results from the Friedman test were followed by Dunn's post‐hoc test for pairwise comparisons within groups. Comparisons between patient and control groups were performed using the Mann–Whitney *U* test. For remaining figures, a paired Student *t*‐test was used between matched groups. A *p* value < 0.05 was considered statistically significant.

## 3 RESULTS

### 3.1 BL001 treatment reduces inflammation in MDMs and DCs

PBMCs were isolated from blood samples procured from healthy and T1D individuals, with an average age of 33 and 37 years, respectively, a BMI of approximately 23 and 24, and equal sex representation (Table 1). CD14^+^ monocytes were purified from PBMCs and derived either into naïve/primed (M1_0_) or pro‐inflammatory macrophages (M1), and immature (iDC) or mature DCs (mDC).^49^, ^50^ Cell viability was greater than 90% for all groups. We first profiled cell surface markers linked to either a pro‐inflammatory or anti‐inflammatory/tolerogenic phenotype on MDMs and DCs isolated from individuals with T1D, treated or not with the small chemical agonist of LRH‐1/NR5A2, BL001. Markers for which expression was significantly altered were also evaluated in MDMs and DCs isolated from healthy individuals to determine if BL001 treatment could render their expression in T1D immune cells similar to the expression observed in healthy cells. Expression levels of CD14 were similar in both healthy and T1D MDMs (M1 and M1_0_) (Figure 1A). BL001 treatment significantly reduced these levels in both T1D M1 and M1_0_, whereas only M1 derived from healthy individuals showed decreased in these levels following BL001 treatment (Figure 1A). In contrast, CD80 levels were significantly higher in T1D MDMs (M1 and M1_0_) than in healthy individuals. Although BL001 treatment significantly reduced CD80 levels in T1D M1, it did not lower them to the levels observed in healthy M1, where BL001 also led to a reduction in CD80 levels (Figure 1B). BL001 also increased the anti‐inflammatory cell surface marker CD206 expression specifically in T1D M1_0_ (Figure 1C). Interestingly, CD206 levels were much higher in T1D MDMs than in healthy MDMs (Figure 1C). In contrast, expression levels of CD86, CD163, CD200R and CD209 remained relatively constant (Figure 1S1A–D). Agonistic activation of LRH‐1/NR5A2 did not alter the expression of proinflammatory cell surface markers on T1D iDCs (Figures 1D–F and S1E–M). However, it increased the expression of CD36 in T1D iDCs compared with either untreated T1D or healthy iDCs with similar levels (Figure 1G). CD36 is implicated in the clearance of apoptotic cells, inhibiting antigen presentation and DC maturation. In contrast, CD54 and CXCR4, expression levels were blunted in BL001‐treated T1D mDCs compared with untreated cells to levels lower to those found in healthy mDCs (Figure 1D,E). Remarkably, CD54 and CXCR4 levels in healthy mDCs were not altered by BL001 treatment whereas CD25 expression levels were blunted in both healthy and T1D BL001‐treated mDCs (Figure 1D,F). Alterations in the expression of MDM and DC surface markers by BL001 were not influenced by sex (Figure 1A–G).

The reduction of pro‐inflammatory surface markers in MDMs and DCs prompted us to assess whether the pharmacological activation of LRH‐1/NR5A2 would also modulate their cytokine secretion profile, further favouring an anti‐inflammatory environment. We found that BL001 treatment of T1D M1_0_ significantly increased the secretion of angiogenin (ANG) and CCL4 (Figure 1H,I), while in T1D M1, it significantly altered the secretion of 6 cytokines (Figure 1H,I). Noteworthy is the decreased secretion of CXCL6 and CCL13, both of which stimulate the chemotaxis of M1 macrophages.^51^ Additionally, BL001 reduced the secretion of TNFSF14, a member of the TNF family that triggers the NF‐κB signalling pathway, leading to the induction of various chemokines, including CXCL6.^52^ The pharmacological activation of LRH1/NR5A2 also diminished the secretion of macrophage migration inhibitor factor and TNFRSF11 (*a.k.a*. osteoprotegerin, OPG), two cytokines known for their pro‐inflammatory activity. In MDMs from healthy donors, BL001 inhibited the secretion of pro‐inflammatory cytokines (M1_0_: IL‐6 and IL‐5; M1: TNF‐beta, IL‐8 and IL‐7) which differed from the cytokines secreted by T1D MDMs (Figure S2A,B). Of particular interest is the increased secretion of IL‐4 and FGF7 in BL001‐treated healthy M1_0_ and M1, respectively, both of which have been shown to promote and M2 phenotype (Figure S2A,B).^53^, ^54^

Activation of LRH‐1/NR5A2 in T1D iDCs significantly decreased the secretion of several members of the CC‐chemokine subfamily (CCL2, CCL24 and CCL26), all of which play roles in various inflammatory diseases by recruiting leukocytes to sites of inflammation (Figure 1J,K).^55^ Correlating with this decline in chemokine secretion, FGF9, which is known to increase expression of pro‐inflammatory chemokines like CCL2 in the central nervous system^56^ exhibited lower levels in T1D iDCs treated with BL001. In parallel, the release of the pro‐inflammatory cytokines IL‐6, LIF (member of the IL‐6 family), IL‐7 and IL‐8, as well as leptin, HGF, IGFBP1, FLT3 ligand (FLT3L), BDNF and PDGFBB, was reduced in BL001‐treated T1D iDC (Figure 1J,K). Of interest, leptin is known for its role in driving DCs maturation, leading to Th1 priming, while the blockade of HGF is associated with the resolution of the pro‐inflammatory phase, considered a critical step in restoring tissue homeostasis.^57^, ^58^ BL001 also decreased the secretion of the proinflammatory cytokines IL‐7, IL‐8 and IL‐1B by T1D mDCs (Figure 1J,K).

Similar to MDMs from healthy donors, the cytokine secretion profile alterations induced by BL001 in DCs from healthy individuals differed significantly from those in T1D DCs, although some common pro‐inflammatory cytokines (IL‐7, HGF, LIF, IGFBP1 and FLT3L) were decreased in both healthy and T1D iDCs (Figure S2C,D). Interestingly, treatment with BL001 led to an increased secretion of pro‐inflammatory cytokines and chemokines such as IFN gamma, IL‐16 and CCL5 in mDCs from healthy individuals. However, BL001 also stimulated the release of several anti‐inflammatory/tolerising cytokines, including TPO, IL‐13 and FGF4, 6 and 7 (Figure S2C,D).^54^, ^59^, ^60^ This dual pro‐ and anti‐inflammatory response is consistent with previous findings in LPS‐matured DCs from healthy donors, where pharmacological modulation of beta‐catenin exhibited similar effects.^61^

Collectively, our findings indicate that the pharmacological activation of LRH‐1/NR5A2 reduces the expression of key pro‐inflammatory cell surface markers in T1D M1 and mDCs with a concomitant reduction in the secretion of several pro‐inflammatory cytokines/chemokines in T1D M1, mDCs and iDCs. Moreover, LRH‐1/NR5A2 activation also imposes an anti‐inflammatory and regenerative phenotype on naïve T1D M1_0_ and iDCs, as assessed by increased secretion of the pro‐angiogenic cytokines ANG and CCL4 in M1_0_ and decreased secretion of the anti‐angiogenic factor HGF in iDCs, further limiting the recruitment/activation of additional M1 and mDCs. Our findings also underscores distinct responses of MDMs and DCs from healthy individuals compared with those with T1D when treated with BL001 underscoring the multifaceted role of LRH1/NR5A2 activation in shaping the immune microenvironment towards an anti‐inflammatory and regenerative state pending the health status of the individual.

### 3.2 LRH‐1/NR5A2 agonistic activation attenuates the pro‐inflammatory genetic signature of T1D MDMs

**FIGURE 2:**
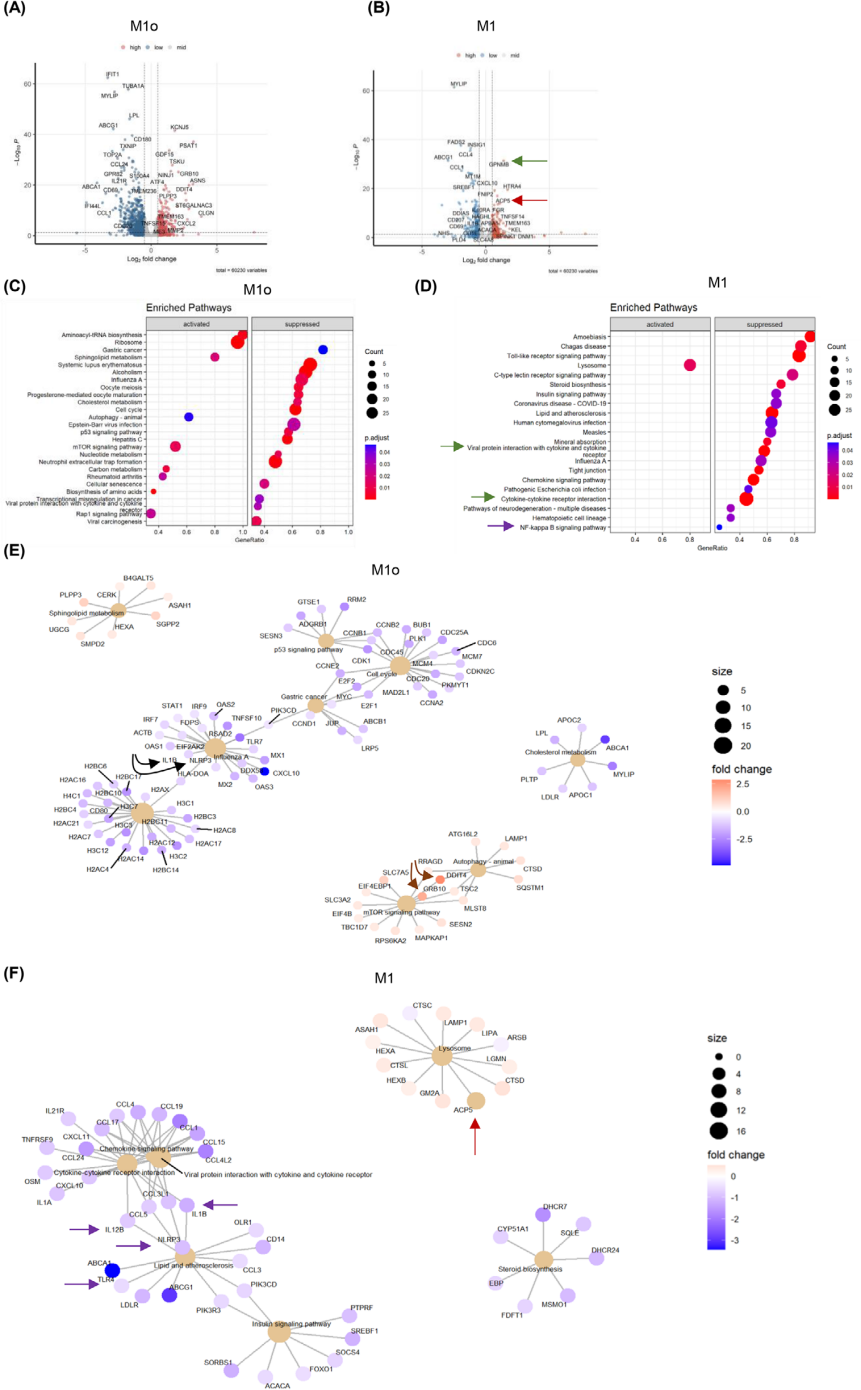
LRH‐1/NR5A2 agonism mitigates the pro‐inflammatory genetic program in T1D monocyte‐derived macrophages (MDM). Volcano plot of differentially expressed genes (DEGs; *p* value < 0.05) in BL001‐treated versus untreated (A) M1_0_ (*n* = 6 independent donors) and (B) M1 (*n* = 6, independent donors). Dot plot of KEGG pathways enriched in BL001‐treated (C) M1_0_ and (D) M1. Cnetplots of selected KEGG pathways of (E) M1_0_ and (F) M1. Arrows highlight genes of interest which are described in the results.

To investigate the molecular consequences resulting from LRH‐1/NR5A2 pharmacological activation, we conducted RNAseq analysis on MDMs obtained from healthy donors and individuals with T1D. While the number of differentially expressed genes (DEGs) in response to BL001 was similar between healthy M1_0_ and M1 (855 vs. 886), T1D M1_0_ exhibited a significantly higher number of DEGs compared with T1D M1 following BL001 treatment (1376 vs. 671) (Figures 2A,B and S3A,B). A comparison of DEGs between healthy and T1D BL001‐treated M1_0_ and M1 macrophages showed minimal overlap of common DEGs (Figure S3C,D) resulting in distinct KEGG enrichment pathways being affected by BL001 (Figures 2C,D and S3E,F) further emphasising that the molecular alterations induced by the pharmacological activation of LRH‐1/NR5A2 are dependent of the health status. Consequently, we focused on assessing the impact of BL001 on T1D MDMs, considering that future clinical trials would target this specific group, including a phase 1 assessment of toxicity as healthy individuals would react differently. GSEA revealed that common DEGs shared by T1D M1_0_ and M1 subpopulations clustered into KEGG pathways predominantly related to hematopoietic cell lineage commitment, cholesterol and fatty acid metabolism. In contrast, DEGs specific to M1_0_ clustered into the cell cycle, genome dynamic and RNA metabolism, whereas genes altered only in M1 clustered into inflammatory pathways and apoptosis (Figure S4). Accordingly, functional enrichment analysis of DEGs in BL001‐treated M1_0_ compared with untreated cells revealed the activation of enriched pathways for ribosomes, sphingolipid metabolism and autophagy.

In contrast, cancer‐related pathways (gastric cancer, p53 and transcriptional misregulation in cancer) and the cell cycle pathway were suppressed, as evidenced by decreased expression of numerous histone‐encoding genes (Figure 2C,E). Of particular interest was the up‐regulation of DDIT4 and GRB10, both of which inhibited mTOR (Figure 2E, brown arrows).^62^, ^63^ DDIT4 conveys the anti‐inflammatory effects of IL‐10 on pro‐inflammatory M1 macrophages through the inhibition of mTOR and the activation of autophagy/mitophagy, resulting in the clearance of damaged mitochondria, lower levels of reactive oxygen species and blunted NLRP3 inflammasome activation.^63^ In line with these effects, transcript levels of NLRP3 and its downstream target IL‐1B were reduced in BL001‐treated M1_0_ (Figure 2E, black arrows). These results suggest that BL001 coerces M1_0_ into an anti‐inflammatory and non‐proliferative phenotype, leading to the secretion of higher levels of regenerative factors (Figure 1I). Notably, in BL001‐treated T1D M1, lysosome was the only positively enriched pathway. In contrast, many pro‐inflammatory‐associated pathways, such as the chemokine signalling pathway, cytokine‐cytokine receptor interaction and Toll‐like receptor (TLR) signalling, were suppressed (Figure 2D). Lysosomal dysfunction has been linked to impaired autophagy flux, contributing to M1 macrophage polarisation under diabetic conditions.^64^ This suggests that the activation of the lysosomal pathway, which includes the ACP5 gene that was significantly up‐regulated in BL001‐treated T1D M1 (Figure 2B,F, red arrows), may be involved in suppressing the pro‐inflammatory phenotype. Primary murine macrophages lacking ACP5 display a pro‐inflammatory phenotype with increased secretion of IL‐1B and IL‐12.^65^ These cytokines and the inflammasome sensor NLRP3, TLR4 and NF‐κΒ signalling pathways were supressed in BL001‐treated T1D M1 (Figure 2D,F, purple arrows).

In light of the transcriptomic effects of BL001, including its inhibition of pro‐inflammatory genes and pathways, we next compared and contrasted DEGs from BL001‐treated versus untreated M1 with those derived from an analysis we performed on M2 versus M1 using RNAseq datasets (GSE24317 and GSE228087) procured from public domains (Figure S5A). This comparison aimed to identify DEGs enriched in M2 that were also altered in BL001‐treated M1 cells, providing insights into the potential of BL001 to reprogram M1 cells towards an anti‐inflammatory M2 phenotype. Such analysis revealed that 30% (234 of 778 transcripts) and 42% (285 of 681 transcripts) of up‐ and down‐regulated genes in BL001‐treated M1 were common with a M2 associated transcript signature (Figure S5A, red circle and Table S1 for gene list). However, the levels of BL001‐mediated regulation of these common DEGs are weaker compared with the transition from a M1 to M2 phenotype extrapolated from datasets indicative of only a partial BL001‐mediated M1 to M2 phenotypic switch (Figure S5B).

### 3.3 LRH‐1/NR5A2 activation inhibits a subset of mitochondrial proteins in T1D M1_0_ and M1

**FIGURE 3:**
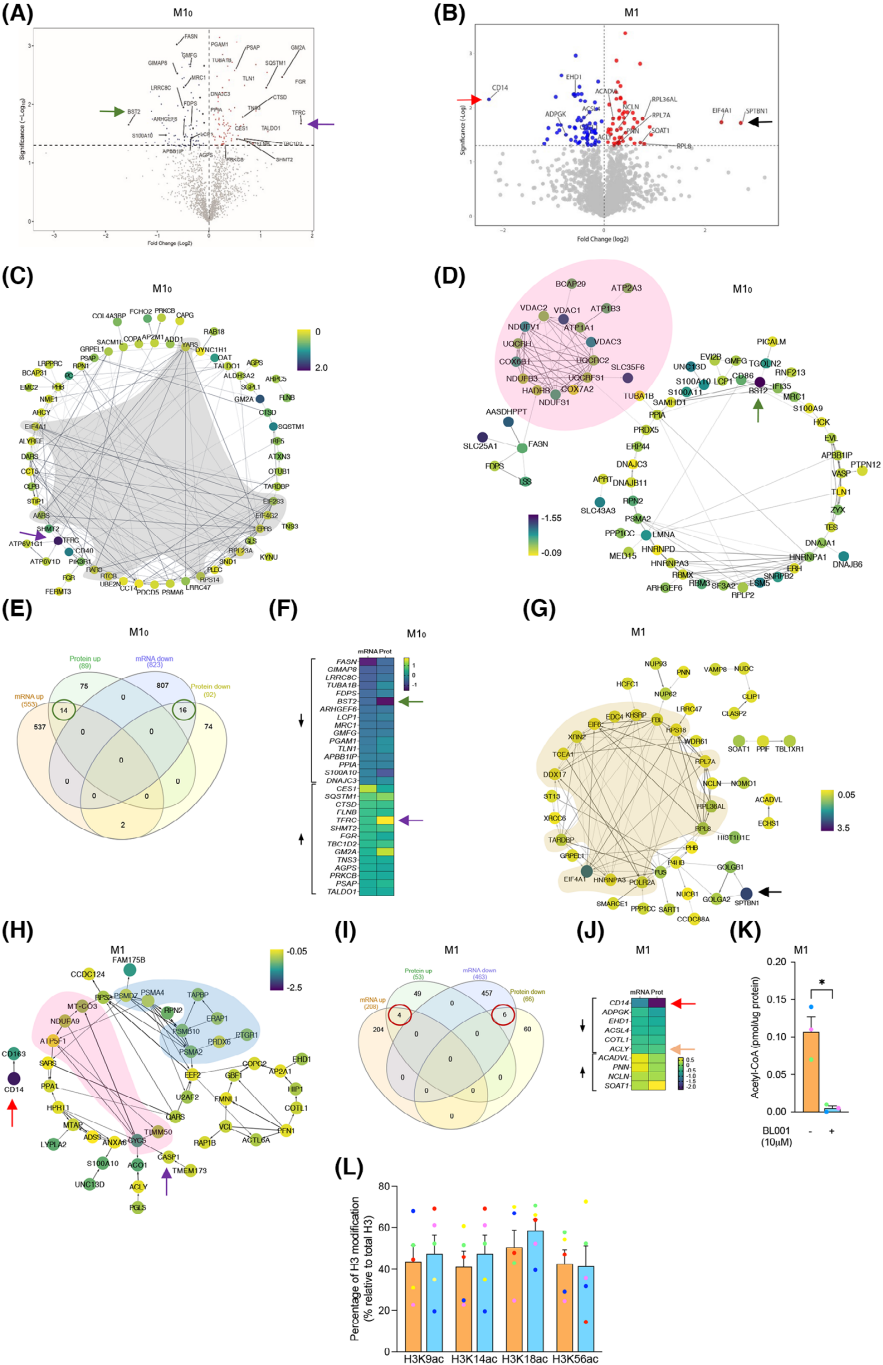
Proteomic alterations induced by LRH‐1/NR5A2 activation in T1D monocyte‐derived macrophages. Volcano plot displaying the most significantly differentially expressed proteins (DEPs, *p* value < 0.05) in (A) BL001‐treated versus untreated M1_0_, derived from *n = 3* independent donors and (B) BL001‐treated versus untreated M1, from *n* = 3 independent donors. Arrows point to genes of interest which are described in the results section. Cytoscape circular layout of significantly (C) up‐regulated proteins and (D) down‐regulated proteins in BL001‐treated M1_0_ compared with untreated controls. RNA‐associated proteins are highlighted within the grey‐shaded area while mitochondrial proteins are emphasised by the pink‐shaded area. (E) InteractiVenn diagram of differentially expressed transcripts/proteins that are significantly altered in either the RNAseq or proteomic analysis of BL001‐treated versus untreated M1_0_. (F) Heatmap of differentially expressed transcripts/proteins common to both the RNAseq and proteomic analysis in BL001‐treated M1_0_ versus untreated M1_0_ marked by green circles in (E). Cytoscape circular layout of significantly (G) up‐regulated proteins and (H) down‐regulated proteins in BL001‐treated M1 as compared with untreated M1. Proteins involved in transcriptional/translational processes are within the French beige‐shaded area while mitochondrial proteins are highlighted in the pink shaded area and proteasome‐associated proteins are in the blue‐shaded area. (I) InteractiVenn diagram of differentially expressed transcripts/proteins that are significantly altered in either the RNAseq or proteomic analysis of BL001‐treated versus untreated M1. (J) Heatmap of differentially expressed transcripts/proteins common to both RNAseq and proteomic analysis of BL001‐treated M1 versus untreated M1_0_ (red circles in I). Arrows point to genes of interest which are described in the results section. (K) Bar graph representing acetyl CoA levels in M1 treated or not with BL001 for *n* = 3 independent donors colour coded and each performed in triplicate. Data are presented as means ± SEM. Student *t*‐test *p < 0.05 as compared with untreated. (L) H3 acetylation levels in M1, treated or not with BL001, were assessed in five independent donors (colour coded). Data are presented as the mean ± SEM, expressed as a percentage relative to total H3 levels.

To map global changes, including post‐transcriptional/translational alterations induced by LRH‐1/NR5A2 activation, we determined the proteomic profile of T1D M1_0_ and M1 treated or not with BL001. In view of differences between healthy and T1D MDMs, we focused only on the latter macrophages. This analysis revealed the presence of 188 DEPs (87 up‐ and 91 down‐regulated proteins, *p* < 0.05) out of a total of 1890 quantifiable proteins in M1_0_ (Figure 3A and Tables S2 and S3). Similarly, in T1D M1, we identified 287 DEPs (151 up‐ and 136 down‐regulated proteins, *p* < 0.05) out of 1953 quantifiable proteins (Figure 3B and Tables S4 and S5). The relatively limited number of DEPs identified posed a challenge to analysing enriched pathways. Consequently, we investigated the PPI network among these DEPs. Consistent with the enrichment of the aminoacyl‐tRNA biosynthesis and ribosome pathways observed in GSEA (Figure 2C), several interacting proteins among the up‐regulated DEPs in BL001‐treated T1D M1_0_ were related to translation initiation and tRNA synthesis (Figure 3C, grey shaded area). Remarkably, a PPI cluster of mitochondrial proteins was identified in the down‐regulated DEPs of BL001‐treated M1_0_, which was not determined by the GSEA (Figure 3D, pink shaded area). Next, we performed an integrative analysis of the transcriptome and proteome to highlight common DEGs and DEPs. This analysis revealed 14 up‐ and 16‐down regulated transcripts/proteins shared by both omics (Figure 3E, green circles and F). Of particular interest, the transferrin receptor (TFRC), common to both omics, was the most up‐regulated DEP in BL001‐treated M1_0_ (Figure 3A,C,F, purple arrow). Deleting this receptor in murine macrophages promoted an M1‐like polarisation driven by IFNγ.^66^ Similarly, BST2, an anti‐viral agent that induces pro‐inflammatory gene expression via NF‐κB,^67^ was the most down‐regulated DEP, consistent with a significant decrease in transcript levels in BL001‐treated M1_0_ (Figure 3A,D,F, green arrow).

In contrast to the GSEA in BL001‐treated M1, which revealed lysosome as the only activated pathway (Figure 2D), most of the up‐regulated DEPs interacting with each other clustered in global gene transcription/translation, including RNA polymerase (POLR2A), translation initiation factors (EIF4A1, the second most up‐regulated DEP) and ribosomal subunits (RPL8, RPL7A, etc.) (Figure 3G, French beige shaded area). The most up‐regulated DEP, SPTBN1, was shown to inhibit inflammatory responses and hepatocarcinogenesis in hepatocellular carcinoma via down‐regulation of the NF‐κB signalling pathway, which was also suppressed in T1D M1^68^ (Figure 3B,G, black arrow and Figure 2D). CD14 was the most down‐regulated DEPs in BL001‐treated M1, aligning with the flow cytometry results (Figure 3B,H,J, red arrow and Figure 1A).^69^, ^70^ Several proteasome‐associated proteins, for which inhibition results in a conversion to an anti‐inflammatory phenotype, were also down‐regulated (Figure 3H, blue shaded area).^71^ Similar to M1_0_, several mitochondrial proteins were repressed in BL001‐treated M1, evidencing that LRH‐1/NR5A2 activation affects mitochondrial function (Figure 3H, pink shaded area).^17^, ^72^ We then compared and contrasted the transcriptome and proteome of BL001‐treated M1, finding that four up‐ and six down‐regulated genes were common to both omics approaches (Figure 3I, red circles and J). The most common down‐regulated gene/protein was CD14^73^ (Figure 3J, red arrow). Of particular interest, ATP‐citrate lyase (ACLY) previously shown to be activated by TLR—leading to increased histone acetylation and the induction of inflammatory genes^74^—was consistently decreased in both omics analyses (Figure 3J, orange arrow). Although BL001‐treated M1 cells exhibited lower Acetyl CoA levels consistent with lower ACLY levels, Histone 3 (H3) acetylation, a key epigenetic marker for the activation of LPS‐induced pro‐inflammatory genes, remained unchanged by the treatment (Figure 3K,L). These results collectively suggest that BL001 inhibits CD14 expression, which in turn blocks LPS binding and activation of the TLR signalling pathway (Figure 2D). This inhibition reduces ACLY activity and Acetyl CoA levels, thereby dampening further histone acetylation and limiting the expression of pro‐inflammatory genes.

### 3.4 LRH‐1/NR5A2 activation drives mitohormesis to enforce a pro‐inflammatory resistant state in T1D M1

**FIGURE 4:**
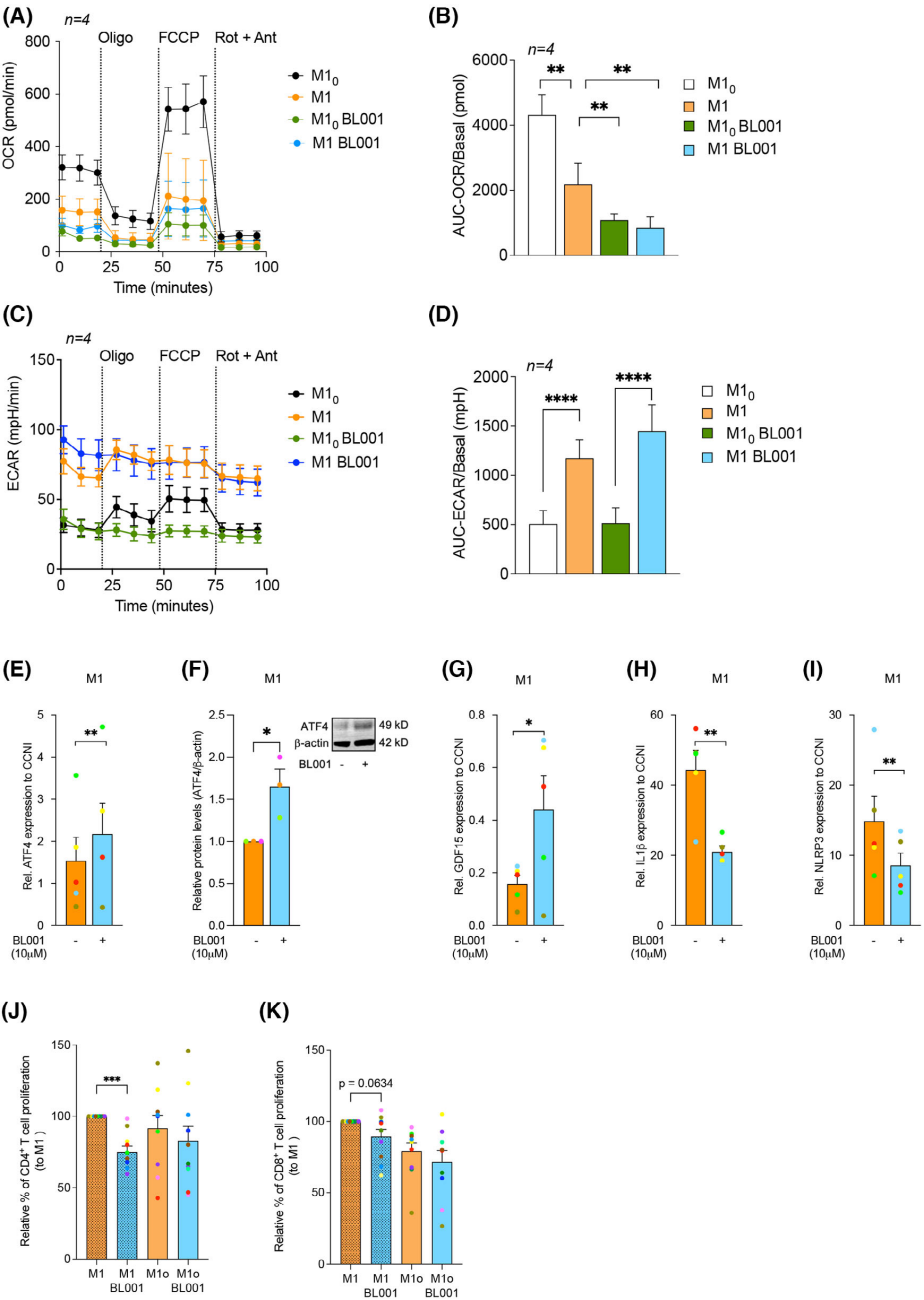
LRH‐1/NR5A2 activation stimulates mitohormesis to enforce LPS‐tolerance in T1D monocyte‐derived macrophages. A mitochondrial stress test was performed on T1D M1_0_ and M1 (LPS/IFNg‐treated M1_0_) treated with or without BL001. Oxygen consumption rate (OCR) (A) profiles and (B) calculated basal OCR. Extracellular acidification rate (ECAR) (C) profiles and (D) calculated basal ECAR. Oligo, oligomycin; FCCP, carbonyl cyanide‐p‐trifluoromethoxyphenylhydrazone; Rot, rotenone; Ant, antimycin A. Paired Student *t*‐test **p < 0.01 ****p < 0.0001. *n* = 4 independent donors. (E) Transcript levels of the mitohormesis‐associated gene ATF4, in BL001 treated or not M1 cells from T1D donors, normalised to the housekeeping gene CCNI (https://housekeeping.unicamp.br/). *n* = 5 independent colour‐coded donors. (F) Protein expression levels of ATF4 in BL001 treated or not M1 cells from T1D individuals and normalised to the housekeeping protein b‐actin. *n* = 3 independent colour‐coded donors. The figure includes a representative western blot image. Transcript levels of (G) GDF15, (H) IL‐1b and (I) NLRP3 in T1D M1 treated with or without BL001. Transcript levels were normalised to the housekeeping gene CCNI (https://housekeeping.unicamp.br/). *n* = 5 independent colour‐coded donors. Data are presented as means ± SEM. Paired Student *t*‐test *p < 0.05, **p < 0.01 as compared with untreated cells. Relative proliferation of autologous (J) CD4^+^ and (K) CD8^+^ T cells in response to co‐culture with T1D MDMs treated or not with 10 µM BL001. *n* = 10 independent colour‐coded donors. Paired Student *t*‐test ***p < 0.0001 as compared with untreated M1.

A hallmark of LPS‐induced pro‐inflammatory reprogramming of macrophages is a time‐dependent shift in ATP production from oxidative phosphorylation (OXPHOS) to glycolysis.^75^ Due to these metabolic changes and as a feedback mechanism to prevent cell impairment, macrophages trigger a stress response called mitohormesis.^76^ This response, which includes a cross‐talk between the nuclei and the mitochondria, attempts to re‐establish mitochondrial homeostasis and induce an LPS tolerance state, thereby avoiding an exacerbated and long‐term pro‐inflammatory response.^77^ As LRH‐1/NR5A2 pharmacological activation suppressed the expression of mitochondrial proteins in M1_0_ and M1, we wondered whether BL001 could precipitate mitohormesis in macrophages and thus trigger an early tolerance state. To test this hypothesis, we assessed the mitochondrial metabolic flux in T1D MDMs. The addition of LPS to M1_0_ to generate M1 significantly reduced the basal as well as maximum OCR with a concomitant increase in basal ECAR as compared with non‐LPS‐treated M1_0_, consistent with the switch from OXPHOS to glycolysis to support a pro‐inflammatory phenotype (Figure 4A–D). BL001 further decreased the basal but not the maximal OCR in M1 and M1_0_, even to lower levels than those found before the LPS treatment (Figure 4A,B). Although not significant, the ECAR was slightly increased in BL001‐treated M1, but not in M1_0_, consistent with decreased OCR (Figure 4C,D). Accordingly, transcript and protein levels of ATF4, the critical activator of mitohormesis and transcript levels of its downstream target GDF15 were significantly increased (Figure 4E–G). In contrast, IL‐1B and the inflammasome sensor NLRP3 expression levels were blunted in BL001‐treated T1D M1 (Figure 4H,I). Consistent with the inhibition of the NLRP3‐inflammasome, protein levels of caspase‐1 (CASP1), a downstream target responsible for IL‐1B activation,^78^ were also reduced in BL001‐treated M1 cells (Figure 3H, purple arrow). Taken together, these results suggest that LRH‐1/NR5A2 agonistic activation triggers mitohormesis in T1D M1, that contributes to a pro‐inflammatory immune‐paralysed state with simultaneous inhibition of the inflammasome, blunting further activation of cytokines.

We next assessed whether this immune‐paralysed state could impact stimulation of naïve CD4^+^ and CD8^+^ T‐cell proliferation, which is typically triggered by professional antigen‐presenting cells such as macrophages and DCs.^79^, ^80^ CD4^+^, but not CD8^+^ T‐cell proliferation, was significantly inhibited by BL001‐treated M1 derived from T1D individuals, indicating that BL001 reprogramming of M1 partly inhibits T‐cell proliferation (Figure 4J,K).

### 3.5 NR5A2 promotes mitochondrial turnover, favouring tolerisation of T1D mDCs

**FIGURE 5:**
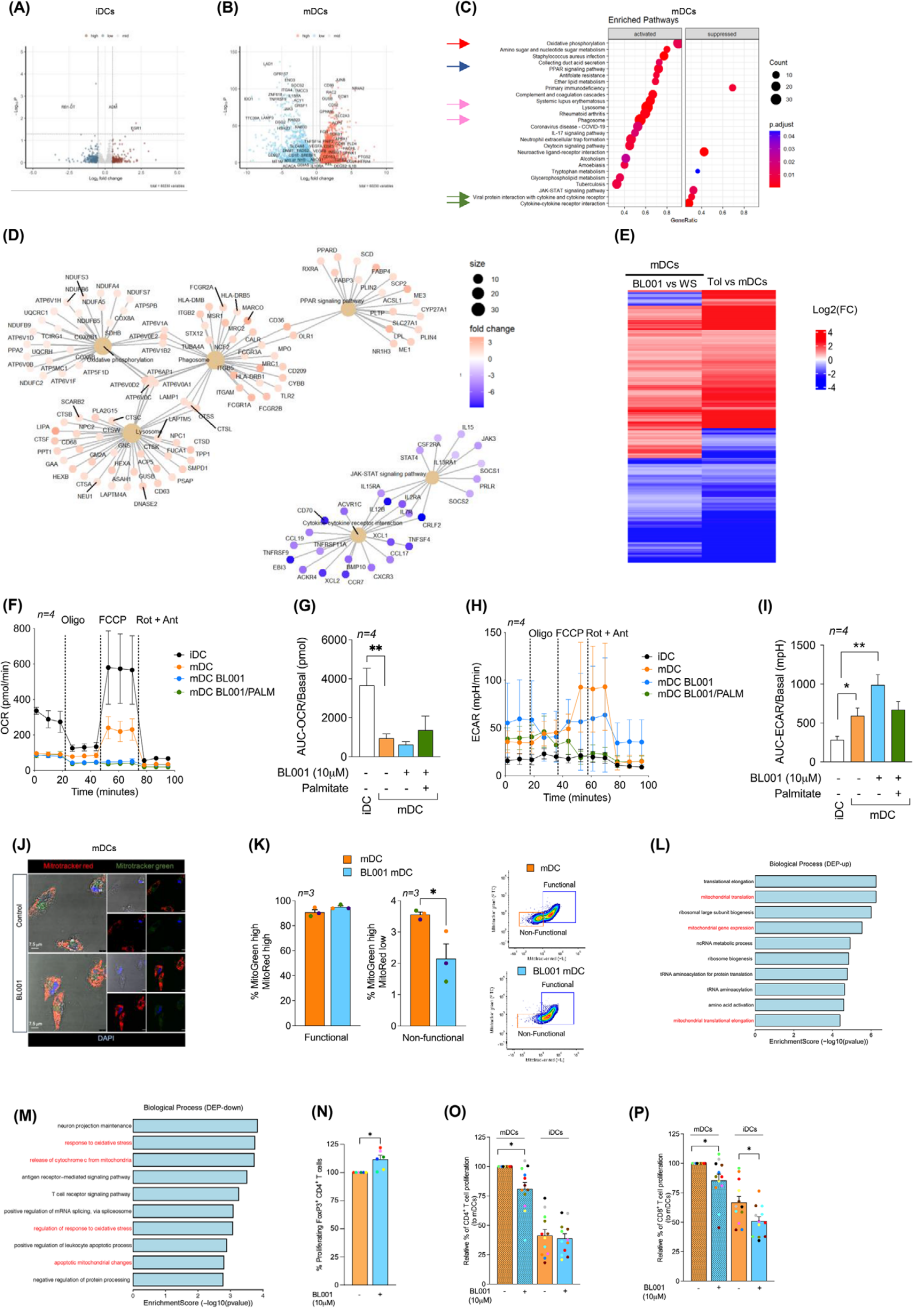
LRH‐1/NR5A2 promotes a tolerogenic phenotype to T1D mDC, suppressing autologous cytotoxic T‐cell proliferation. Volcano plot of DEGs in BL001‐treated versus untreated (A) iDCS (*n* = 5 independent donors) and (B) mDCs (*n* = 4 independent donors). (C) Dot plot of KEGG pathways enriched in BL001‐treated mDCs. Arrows point to pathways of interest which are described in the results section. (D) Cnetplots of selected KEGG pathways modulated by BL001 in mDCs. (E) Log_2_(FC) heatmap of common DEGs between mDC from T1D individuals after BL001 treatment (*p* value < 0.05) and in IL‐10‐induced TolDC versus mDC (GSE180761; padj < 0.05). (F and G) Oxygen consumption rate (OCR) profiles and calculated basal OCR as well as (H and I) extracellular acidification rate (ECAR) profiles and calculated basal ECAR of T1D iDC and mDC treated with or without BL001 and with palmitate. Oligo, oligomycin; FCCP, carbonyl cyanide‐p‐trifluoromethoxyphenylhydrazone; Rot, rotenone; Ant, antimycin A. *n* = 4 independent donors (colour matched). Paired Student *t*‐test *p < 0.05 and **p < 0.01 as compared with iDCs. (J) Representative confocal immunofluorescence images of cells labelled with MitoTracker green and MitoTracker red for mitochondria, with nuclei counterstained using DAPI. (K) Bar chart quantification for mitochondrial functionality, as determined by flow cytometry based on MitoTracker green and MitoTracker red. Representative flow cytometry plots are provided for illustration. *n* = 3 independent colour‐code donors. Paired Student *t*‐test *p < 0.05 as compared with untreated mDC. Bar plot ranking of the top ten GO biological process terms associated with (L) up‐regulated and (M) down‐regulated proteins in BL001‐treated mDCs (*p* value < 0.05) (N) Relative autologous proliferation of FoxP3^+^/CD4^+^ in the presence of T1D mDCs treated with or without 10 µM BL001. Paired Student *t*‐test **p < 0.01 as compared with untreated. Relative autologous proliferation of (O) CD4^+^ and (P) CD8^+^ T‐cells in the presence of T1D DCs treated with or without 10 µM BL001. Data are presented as means ± SEM. Paired Student *t*‐test *p < 0.05 as compared with untreated cells.

Having defined the molecular mode of action of LRH‐1/NR5A2 in macrophages, we next focused on the genetic adaptations induced by BL001 in DCs. RNAseq analysis revealed that BL001 altered the expression of only a few genes in T1D iDCs, with no significant changes detected in either KEGG pathways or GO enrichment terms (Figure 5A). In contrast, treatment with BL001 altered the expression of 672 transcripts in iDCs derived from healthy individuals, with no overlap to those changed in BL001‐treated T1D iDCs (Figure S6A,C). Notably, OXPHOS emerged as the most suppressed KEGG pathway in BL001‐treated healthy iDCs (Figure S6E). Similar to MDMs, these results underscore fundamental molecular differences between healthy and T1D iDCs. BL001 treatment altered the expression of 8339 and 3590 transcripts in healthy and T1D mDCs, respectively (Figures 5B and S6B). The majority of BL001‐modulated DEGs in T1D mDCs overlapped with those in healthy mDCs, resulting in both cell types sharing similar enriched pathways (Figures S6D,F and 5C). However, given the larger number of DEGs affected by BL001 in healthy mDCs and the distinct genetic profiles between BL001‐treated iDCs from healthy and T1D individuals, we focused on the molecular characterisation of T1D mDCs. This focus was driven by the rationale that future clinical trials will primarily target this group. Functional enrichment analysis of BL001‐treated T1D mDCs revealed the activation of the lysosomal pathway and suppression of the ‘viral protein interaction with cytokine and cytokine receptor’ and ‘cytokine and cytokine receptor interaction’ pathways, which were also altered in T1D M1 treated with BL001 (Figures 5C,D and 2D, green arrows). The most activated pathway in BL001‐treated T1D mDCs was OXPHOS (Figure 5C, red arrow and D). Like macrophages, switching from OXPHOS towards glycolysis is the main trigger activating mDCs.^81^ Thus, stimulation of OXPHOS via increased fatty acid oxidation driven by the PPAR signalling pathway (also among the top activated pathways—Figure 5C blue arrow) may induce an anti‐inflammatory and tolerogenic phenotype in mDCs. To substantiate the latter, we compared and contrasted DEGs from BL001‐treated versus untreated T1D mDCs with those derived from an analysis we performed using a RNAseq dataset procured from a public domain (GSE117945) in which mDCs were polarised towards a TolDCs phenotype using IL‐10.^82^ This comparison aimed to identify DEGs enriched in IL‐10‐induced TolDCs that were also altered in BL001‐treated mDCs cells, providing insights into the potential reprogramming effects of BL001. Such analysis revealed that 48% of up‐regulated (1240 of 2788 transcripts) and 45% of down‐regulated (1021 of 2122 transcripts) of DEGs in BL001‐treated mDCs overlapped with a TolDC signature induced by IL‐10 (Figure 7S). Furthermore, the levels of BL001‐mediated regulation of these common DEGs were as strong as IL‐10 supporting the concept that BL001 induces a TolDCs phenotype to mDCs (Figure 5E). We next assessed the mitochondrial metabolic flux of DCs isolated from T1D individuals treated with or without BL001 and palmitate to ascertain whether stimulation of the OXPHOS pathway was promoting a tolerogenic phenotype to mDCs. Similar to MDM, maturation/activation of iDCs resulted in decreased basal and maximal OCR with a concomitant increase in basal ECAR (Figure 5F–I). The addition of BL001 alone or together with palmitate did not increase basal OCR compared with untreated mDCs whereas basal ECAR was further increased by BL001 (Figure 5F–I). Remarkably, BL001 treatment completely abrogated maximal respiration in mDCs, indicating a potential shutdown and turnover of the mitochondria network. The latter is consistent with the up‐regulation of the lysosomal and phagosome pathways in BL001‐treated mDCs, along with genes associated with increased acidification and hydrolase activity (Figure 5C, pink arrows and D) that are implicated in mitophagy to eliminate dysfunctional mitochondria (Figure 5D). To corroborate this premise, mitochondrial mass and membrane potential were assessed by flow cytometry using the probes MitoTracker Green (binding covalently to mitochondrial proteins, thus providing an assessment of mass) and MitoTracker Red (taken up by polarised mitochondria, hence gauging function).^46^ BL001‐treated T1D mDCs displayed a reduced proportion of cells with non‐functional mitochondria (MitoTracker Green^High^ and MitoTracker Red^low^) compared with untreated cells (Figure 5J,K). Next, comparative proteomic profiling was conducted on BL001‐treated mDCs versus vehicle‐treated cells to assess whether DEPs associated with mitochondrial biogenesis were increased, correlating with higher expression levels of mitochondrial‐related genes (Figure 5D). Two hundred and forty‐five DEPs (201 up‐ and 41 down‐regulated proteins, *p* < 0.05) were identified out of a total of 6181 quantifiable proteins in mDCs (Tables S6 and S7). Functional classification through GO analysis demonstrated that a significant portion of up‐regulated DEPs were enriched in biological processes related to mitochondrial homeostasis—turnover, biogenesis and function (Figure 5L red biological processes and Table S8). In parallel, down‐regulated DEPs were segregated into several GO biological processes linked to oxidative stress and mitochondria death (Figure 5M, red biological processes). Taken together, these results support the premise that LRH‐1/NR5A2 activation in mDCs facilitates mitochondrial turnover, which is associated with the emergence of a tolerogenic phenotype (Figure 5E). Consistent with this, BL001‐treated mDCs facilitated the expansion of an autologous Foxp3^+^/CD4^+^ regulatory T‐cell subpopulation while simultaneously inhibiting the proliferation of the global CD4^+^ and CD8^+^ cytotoxic T‐cells in individuals with T1D (Figure 5N,O). Furthermore, BL001‐treated T1D iDCs also displayed reduced capacity to stimulate CD8^+^ T‐cell proliferation, which correlated with increased levels of CD36 in these cells (Figures 5P and 1G).

### 3.6 BL001 promotes anti‐inflammatory T‐cell dynamics and enhances regulatory T‐cell subpopulations in T1D PBMCs

**FIGURE 6:**
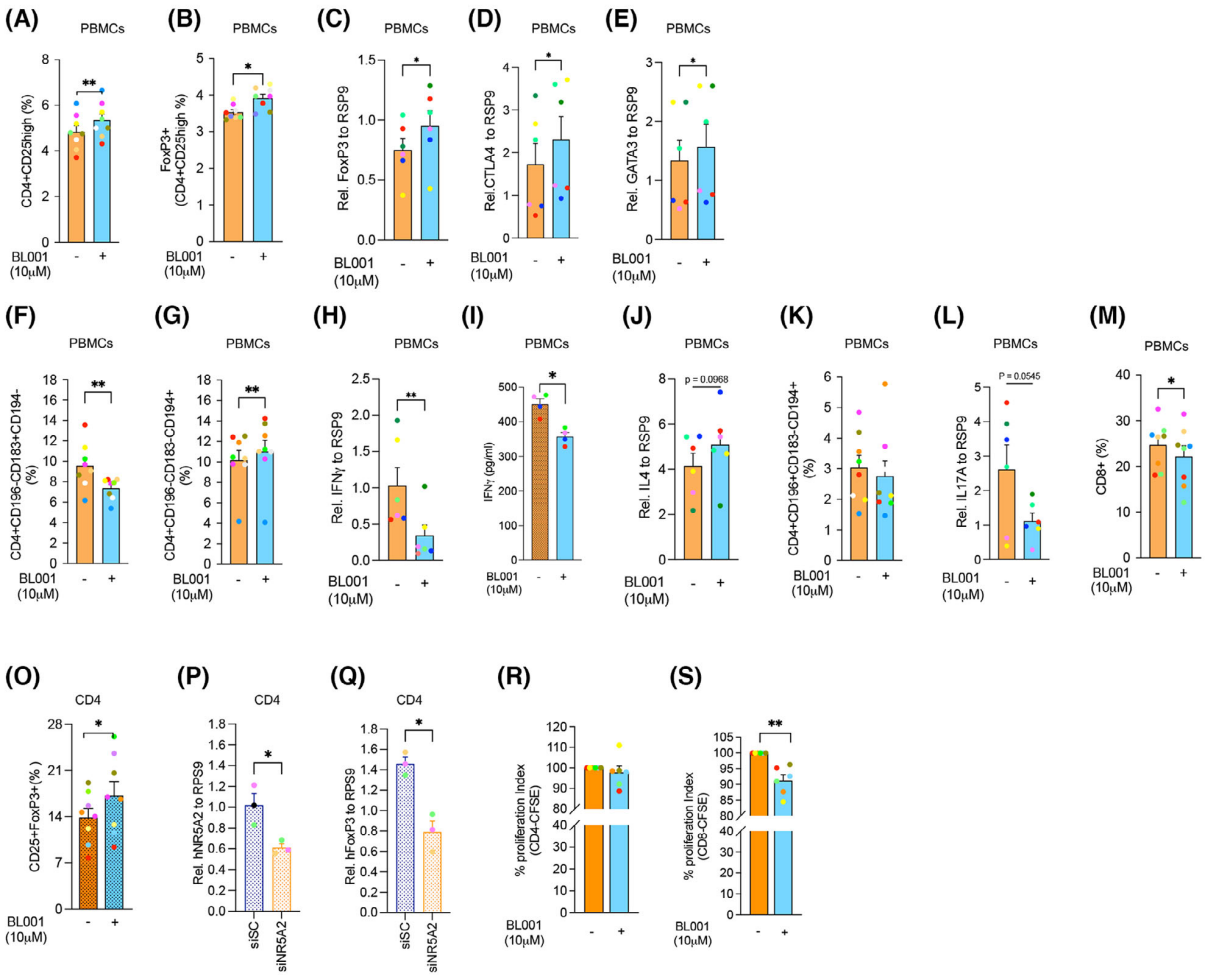
LRH‐1/NR5A2 agonism alters CD4^+^ and CD8^+^ T‐cell subpopulations in T1D individuals. PBMCs were purified from individuals with T1D and exposed to 10 µM BL001 every 24 h for a total duration of 48 h with a final dose given 30 min prior to analysis. Flow cytometry immunophenotyping of (A) CD4^+^CD25^+^ and (B) Tregs; CD4^+^CD25^+^FoxP3^+^ cell subpopulations. The initial gating defining the CD4 population was always set at 10 000 cells. *n* = 8 independent individuals with T1D. Relative transcript levels of (C) FoxP3, (D) CTLA4 and (E) GATA3. Data were normalised to the housekeeping gene RSP9. *n* = 5 T1D independent donors. Flow cytometry immunophenotyping of (F) Th1; CD4^+^CD196^−^CD183^+^CD194^−^ and (G) Th2; CD4^+^CD196^−^CD183^−^CD194^+^. The initial gating defining the CD4 population was always set at 10000 cells. *n* = 8 independent individuals with T1D. Relative (H) transcript and (I) secreted levels of IFNγ as well as of (J) IL‐4. Transcript levels of IFNγ were normalised to the housekeeping gene RSP9. *n* = 4–6 T1D independent donors. (K) Th17/22; CD4^+^CD196^+^CD183^−^CD194^+^ immunophenotyping and (L) IL17 transcript levels normalised to the housekeeping gene RSP9. *n* = 6–8 T1D independent donors. (M) CD8^+^ immunophenotyping. *n* = 8 independent individuals with T1D. (O) CD4^+^ cells were isolated from PBMCs and treated with BL001 as described above. Cells were then analysed by flow cytometry for the cell surface markers CD25^+^FoxP3^+^. Relative (P) LRH‐1/NR5A2 and (Q) FoxP3 transcript levels in either siScrambled (siSc) or siNR5A2‐treated PBMCs. Data were normalised to the housekeeping gene RSP9. *n* = 3 T1D independent donors. CD14^−^ PBMCs were labelled with CFSE and stimulated/expanded using antiCD3/CD28 before the addition of CD4^+^ T‐cells (at a 1:2 ratio, respectively, from the same donor). Proliferation of (R) CD4^+^/CFSE^+^ and (S) CD8^+^/CFSE^+^ subpopulations was assessed by flow cytometry 4 days post co‐culturing. *n* = 6 T1D independent donors. Each donor is colour coded. Data are presented as means ± SEM. Paired Student *t*‐test *p < 0.05 and **p < 0.01 as compared with untreated cells.

We next assessed whether BL001 could also impart an anti‐inflammatory landscape to T‐cell subpopulations within PBMCs isolated from individuals with T1D, including the expansion of FoxP3^+^ T‐cells, which enforce peripheral self‐tolerance. PBMCs treated with BL001 exhibited a significantly higher number of CD4^+^CD25^+^ and CD4^+^CD25^+^FoxP3^+^ T‐cells than untreated PBMCs (Figure 6A,B). This increase correlated with significantly higher expression levels of FoxP3, CTLA4 and GATA3, three key transcription factors involved in the generation of Tregs (Figure 6C–E). Concurrently, a decrease in the T helper‐1 (Th1) cell population and an increase in the T helper‐2 (Th2) cells were observed, indicating a shift in the helper T‐cell dynamics (Figure 6F and G). Consistent with this shift, IFNγ transcript and secretion were blunted in BL001‐treated PBMCs whereas IL‐4 secretion was mildly increased, albeit the latter not reaching statistical significance (Figure 6H–J). No changes were perceived in the subpopulation of Th17/Th22 while secretion of IL‐17A was moderately blunted by BL001 (Figure 6K,L). The treatment also reduced the CD8^+^ T‐cell subpopulation (Figure 6M). Upon activation of isolated CD4^+^ T‐cells, a significant increase in the CD4^+^CD25^+^FoxP3^+^ T‐cell subpopulation was further observed in the presence of BL001, suggesting that the pharmacological activation of LRH‐1/NR5A2 promotes a regulatory phenotype under conditions of T‐cell activation (Figure 6O). Silencing of LRH1/NR5A2 led to a decrease in FoxP3 levels in CD4^+^ T‐cells, suggesting that the nuclear receptor regulates the expression of FoxP3 in these cells (Figure 6P,Q). Although CD4^+^ T‐cell proliferation showed no significant changes, CD8^+^ T‐cell proliferation was decreased by BL001 treatment of activated T‐cells (Figure 6R,S). These results indicate that LRH‐1/NR5A2 promotes anti‐inflammatory T‐cell dynamics and enhances regulatory T‐cell subpopulations in T1D PBMCs.

### 3.7 BL001 mitigates PBMC‐induced apoptosis and maintains INS expression in hiPSC‐derived islet organoids

**FIGURE 7:**
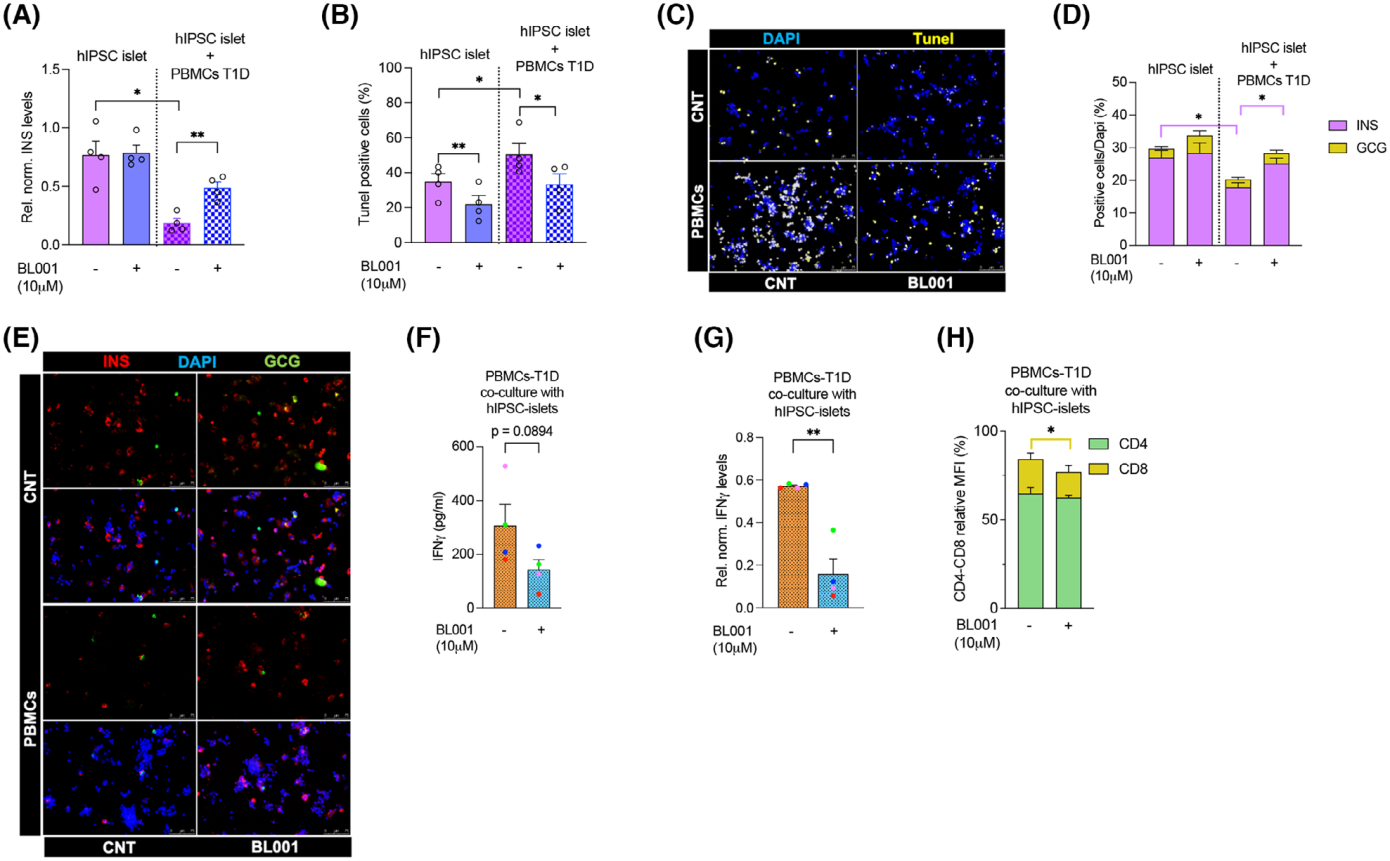
LRH1/NR5A2 activation modulates insulin production, apoptosis and immune response in human iPSCs‐derived islet‐like organoids co‐cultured with PBMCs from T1D individuals. Human IPSCs‐derived islet‐like organoids (hIPSC‐islet) were either cultured alone or co‐cultured with PBMCs from T1D donors, and treated or not with 10 µM BL001. (A) Transcript levels of insulin (INS) in hIPSC‐islets were measured and normalised to the housekeeping gene RPS9. (B) Quantification of TUNEL‐positive cells was determined by immunofluorescence to assess apoptosis in hIPSC‐islets. (C) Representative immunofluorescence images of (B) showing DAPI (blue) and TUNEL (yellow) staining in hIPSC‐islets alone (CNT) or with PBMCs, and treated with BL001 or not (CNT). (D) Stack bar with the quantification of INS (pink) and GCG (yellow) positive areas in hiPSC‐derived islets. Pink error bars refer to INS. (E) Representative immunofluorescence images showing insulin (INS; red), glucagon (GCG; green) and DAPI (blue) staining in hIPSC‐islets alone (CNT) or with PBMCs, and treated with BL001 or not (CNT). (F) IFNγ protein levels measured by BD™ Cytometric Bead Array (CBA) Human Th1/Th2 Cytokine Kit II in the media of PBMCs from T1D individuals co‐cultured with hiPSC islets, treated or not with 10 µM BL001. (G) Transcript levels of IFNγ in PBMCs from T1D individuals co‐cultured with hIPSC‐islets and treated or not with BL001, normalised to the housekeeping gene Cyclophilin. (H) Stack bar with the relative MFI values of CD4^+^ (green) and CD8^+^ (beige) from the PBMCs cultured with hIPSC‐derived islets. Beige error bars refer to CD8. The hIPSC‐derived islet‐like organoids were generated from separate differentiation experiments. Data are presented as means ± SEM from four independent experiments with four different T1D donors colour coded. Statistical significance was determined using a paired Student's *t*‐test, with *p < 0.05 and **p < 0.01 indicating significant differences compared with untreated cells.

Although we defined the mode of action of LRH‐1/NR5A2 agonistic activation in MDMs and DCs, as well as its impact on T‐cells in vitro, the overall physiological effect of BL001 in a more complex environment where these immune cells interact with islets remains unclear. To address this question, we co‐cultured PBMCs isolated from individuals with T1D with hiPSC‐derived islet organoids in the presence or absence of BL001. Since hiPSC‐derived β‐cells obtained from both healthy individuals and those with T1D were shown to be equally sensitive to either cytokines or PBMCs,^83^ we selected the hiPSC‐derived islet organoids model,^32^ in which we previously demonstrated a protective effect of BL001 against cytokine‐induced apoptosis.^20^ Experiments were designed to compare the impact of LRH1/NR5A2 activation on hiPSC‐derived islet organoids, PBMCs and the combination of both. PBMCs were activated to simulate a pro‐inflammatory environment and evaluate whether BL001, alone or in combination with hiPSC‐derived islet organoids, could enhance organoid survival and reduce the secretion of pro‐inflammatory cytokines. BL001 treatment did not significantly alter insulin (INS) mRNA expression as compared with untreated hiPSC‐derived islet organoids (Figure 7A). However, it decreased hiPSC‐derived islet organoid cell apoptosis while maintaining a consistent percentage of INS and glucagon (GCG)‐positive cells (Figure 7B–E). Co‐culture of hiPSC‐derived islet organoids with PBMCs significantly decreased INS transcript levels and the percentage of INS‐positive β‐like cells, in line with the increased apoptosis compared with hiPSC‐derived islet organoids alone. Adding BL001 significantly reversed these effects, restoring levels similar to those observed in untreated hiPSC‐derived islet organoids without PBMCs (Figure 7A–E).

Consistent with our previous results (Figure 6), IFNγ secretion and expression levels were reduced in BL001‐treated co‐cultures of PBMCs with hiPSC‐derived islet organoids, although the former did not reach statistical significance (Figure 7F,G). Coherent with a reduction in CD8^+^ T‐cell subpopulation observed in BL001‐treated PBMCs (Figure 6F), this subpopulation was also significantly reduced in co‐cultures of PBMCs and hiPSC‐derived islet organoids‐treated with BL001 correlating with decreased IFNγ (Figure 7H). Taken together, these results suggest that pharmacological activation of LRH‐1/NR5A2 mitigates PBMCs‐induced β‐cells apoptosis likely via decreased CD8^+^ cytotoxic T‐cells and reduced IFNγ, a key cytokine contributing to the triggering and amplification of autoimmunity.^84^

## 4 DISCUSSION

Despite substantial clinical and research efforts, a long‐term pharmacological solution that can significantly reverse hyperglycaemia in T1D has yet to be discovered. The complexity of T1D highlights the urgent need for a fundamental paradigm shift in our approach to understanding the disease´s intricate mechanisms, which involves a dynamic interaction between the immune system and pancreatic cells.^85^ The ultimate T1D pharmacological therapy should foster the resolution of the autoimmune attack/proinflammatory process rather than suppressing it, leading to cell survival and regeneration similar to wound healing.^9^ Herein, using PBMCs, MDMs, DCs and T‐cells isolated from individuals with established T1D and a combination of cell surface marker analysis, omics profiling and functional studies, we demonstrate that the pharmacological activation of the NR LRH‐1/NR5A2 resolves the pro‐inflammatory environment by (1) locking M1_0_ in a naïve state while suppressing the pro‐inflammatory M1‐like phenotype of M1 through mitohormesis, (2) fostering a reprogramming of mDCs towards TolDCs by promoting mitochondrial turnover, thereby reducing CD4^+^ and CD8^+^ T‐cells proliferation and (3) increasing subpopulations of CD4^+^/CD25^+^/FoxP3^+^ T‐cells as well as Th2 cells, leading to diminished CD8^+^ T‐cell proliferation. Our study also establishes that the activation of LRH‐1/NR5A2 can enhance the survival of hiPSC‐derived islet organoids co‐cultured with PBMCs, preserving INS expression. To the best of our knowledge, our study is one of the first to demonstrate that a small chemical pharmacological compound imparts anti‐inflammatory properties to fully activated M1 and mDCs of individuals with T1D. Most studies have primarily concentrated on iDCs, evaluating the effects of compounds such as 1α,25‐dihydroxyvitamin D3 on DCs maturation and activation,^86^, ^87^ but none have investigated their potential to transform fully mature DCs isolated from individuals with T1D into a tolerogenic phenotype. Consequently, our findings hold significant implications for human clinical studies since reversing T1D will require addressing the chronic pro‐inflammatory/autoimmune responses primarily driven by macrophages and DCs activated by islet self‐antigens. These cells are pivotal in triggering T‐cell proliferation and the subsequent β‐cell destruction.^2^ Our findings also reveal that MDMs and DCs from healthy donors respond differently to the pharmacological activation of LRH‐1/NR5A2 compared with those from individuals with T1D, underscoring fundamental molecular alterations caused by the disease. Therefore, caution should be exercised when designing future Phase 1 clinical trials that are usually conducted in healthy volunteers.

Our findings also emphasise the cell context‐dependent nature of the genetic alterations and phenotypic outcomes induced by LRH‐1/NR5A2 pharmacological activation. This is evidenced by the diverse molecular pathways regulated in BL001‐treated T1D M1 and mDCs, with many pathways suppressed in M1 but activated in mDCs. In M1, LRH‐1/NR5A2 activation suppressed numerous pathways implicated in inflammatory responses (15 out of 20), correlating with decreased expression of key cell surface markers such as CD14 and CD80 and reduced secretion of pro‐inflammatory cytokines and chemokines. Interestingly in T1D M1 cells, BL001 partially induced an M2 genetic signature without increasing M2‐associated surface markers such as CD206, CD209, or CD200R. This suggests that LRH‐1/NR5A2 activation likely suppresses the pro‐inflammatory M1 phenotype, rather than promoting a full transition to an M2‐like phenotype. The concept of M1 incapacitation is further evidenced by the instatement of an immune‐paralysed phenotype via mitohormesis induced by BL001, which is consistent with previous studies reporting that LPS/IFNγ‐mediated mitochondrial shutdown prevents IL‐4 polarisation of M1 towards an M2 phenotype.^88^ Additionally, BL001´s anti‐inflammatory properties are highlighted by the inhibition of multiple enriched pathways related to inflammatory responses (systemic lupus erythematosus, influenza A, Epstein–Barr virus, among others), as well as pathways involved in either cell cycle or cellular senescence in BL001‐treated M1_0._ These molecular events translated to the inhibition of CD4^+^ T‐cell proliferation. Notably, the mitohormesis‐related factor GDF15, which was increased in BL001‐treated T1D M1s, was shown to be expressed in a subset of macrophages and to promote muscle regeneration.^89^ Additionally, GDF15 expression is lower in the T1D pancreas, and its exogenous administration prevents insulitis progression in NOD mice and protects isolated human pancreatic islets.^90^ Concurrently, pharmacological activation of LRH‐1/NR5A2 led to up‐regulated expression of ANG and CCL4 in M1_0_ from T1D individuals. ANG is associated with an anti‐inflammatory and wound‐healing response in macrophages.^91^ CCL4 is recognised as a chemokine‐attracting T‐cell, including Tregs,^92^ but it also promotes angiogenesis via angiopoietin‐2 in oral squamous cell carcinoma.^93^ Taken together, these data suggest that BL001 favours a regenerative environment through increased expression of GDF15, ANG and CCL4 from MDMs.^9^, ^19^ In this context, we have previously shown that CCL4 serum levels were increased in BL001‐treated hyperglycaemic RIP‐B7.1 mice correlating with immune coupled α‐ to β‐cell trans‐differentiation.^19^ ANG was also shown to cleave tRNAs, through its ribonuclease activity, thereby transiently inhibiting cell translation under stress conditions and allowing them to adapt to their new situation.^94^, ^95^ Accordingly, the top two activated enriched pathways in BL001‐treated T1D M1_0_ were aminoacyl‐tRNA biosynthesis and ribosome, evidencing an autocrine effect of ANG to alleviate stress‐induced molecular reprogramming.

In contrast to MDMs, the pharmacological activation of LRH‐1/NR5A2 appears to reprogram T1D mDCs toward a tolerogenic‐like phenotype. This is evidenced by the fact that approximately 48% of DEGs in BL001‐treated mDCs cells overlap with a TolDCs signature, leading to the activation of key molecular pathways associated with immune tolerance, such as OXPHOS), PPAR signalling, lysosomal activity and phagocytosis. Furthermore, levels of several cell surface markers associated with mDCs (CXCR4 and CD54) were decreased, as did the secretion of IL‐8, IL‐7 and IL‐1β in cells treated with BL001. High circulating levels of IL‐8 have been associated with poor metabolic control in adolescents with T1D^96^ while two independent studies have demonstrated that blocking the IL‐7 receptor could reverse autoimmune diabetes in NOD mice by repressing Teffs.^97^, ^98^ A recent clinical trial assessing the efficacy of a humanised anti‐IL‐7R monoclonal antibody in subjects with T1D revealed a significant decline in CD4^+^ and CD8^+^ effector and central memory T‐cells along with an increase in Tregs.^99^ BL001 treatment of mDCs from T1D donors similarly suppressed the proliferation of CD4^+^ and CD8^+^ T‐cells, suggesting a potential role of IL‐7 in this process. This premise is further substantiated by the decreased levels of IL‐7 in BL001‐treated iDCs, which also impeded CD8^+^ T‐cell expansion. Based on the present RNAseq data revealing BL001‐mediated activation of the PPAR and OXPHOS genetic pathways, with no net differences in mitochondrial respiration experiments, we suggest that the phenotypic switch induced by LRH‐1/NR5A2 activation is likely mediated by enhanced mitochondrial turnover. The latter is supported by decreased mDCs with dysfunctional mitochondria correlating with omics data evidencing mitochondrial biogenesis. Previous studies have demonstrated that mitophagy/autophagy contributes to maintaining an anti‐inflammatory phenotype in immune cells.^100^

Our study also underscores the potent immunomodulatory benefits of LRH1/NR5A2 activation on whole PBMCs of individuals with T1D, characterised by an increase in CD4^+^/CD25^+^/FoxP3^+^ T‐cells and Th2 cells with a concurrent decrease in Th1 cells, consistent with our previous findings in mice.^19^ The reduction in FoxP3 expression upon LRH‐1/NR5A2 silencing in PBMCs directly links the observed immunomodulatory effects of BL001 to the activation of the NR and increased FoxP3^+^ T‐cells. BL001 treatment also led to a decrease in the CD8^+^ T‐cell subpopulation, whether cultured alone or co‐cultured with hiPSC‐derived islet organoids, while the CD4^+^ T‐cell subpopulation remained unchanged. This decrease in CD8^+^ T‐cell subpopulation, which was also observed with BL001‐treated mDCs, holds significant therapeutic implications given that CD8^+^ T‐cells are pivotal in beta cell destruction and sustained inflammation in T1D.^101^ In line with this idea, BL001 also prevented PBMCs allogenic‐induced hiPSC‐derived islet organoid cell apoptosis and decreased INS mRNA expression; this correlated with decreased secretion IFNγ, a key cytokine released by cytotoxic CD8^+^ T‐cells that causes β‐cell dysfunction and increased HLA class I expression and chemokine release, thus attracting cytotoxic CD8+ T‐cells and amplifying autoimmunity.^84^ Additionally, IFNγ has been shown to drive the differentiation of monocytes into DCs and MDMs, further perpetuating this autoimmune cycle. By reducing IFNγ levels, BL001 helps restore immune homeostasis and mitigate autoimmunity.

Our results differ from those of Schwaderer and colleagues, who reported that LRH‐1/NR5A2 inhibition, rather than activation, promotes an anti‐inflammatory phenotype in LPS‐activated human PBMC‐derived monocytes, as evidenced by reduced TNF secretion.^102^ We argue that the discrepancies are due to differences in experimental design. In the cited study, the authors pre‐treated healthy monocytes with an LRH‐1/NR5A2 inhibitor before inducing a pro‐inflammatory phenotype with LPS. In contrast, our approach involved inducing the pro‐inflammatory phenotype first, followed by the addition of BL001. This difference in timing likely explains the variation in observed outcomes, as the sequence of treatments may influence how cells respond to LRH‐1/NR5A2 modulation. The latter also reinforces the notion that the role of LRH‐1/NR5A2 in inflammation is highly specific to the tissue, cell type and disease context.^103^

**FIGURE 8:**
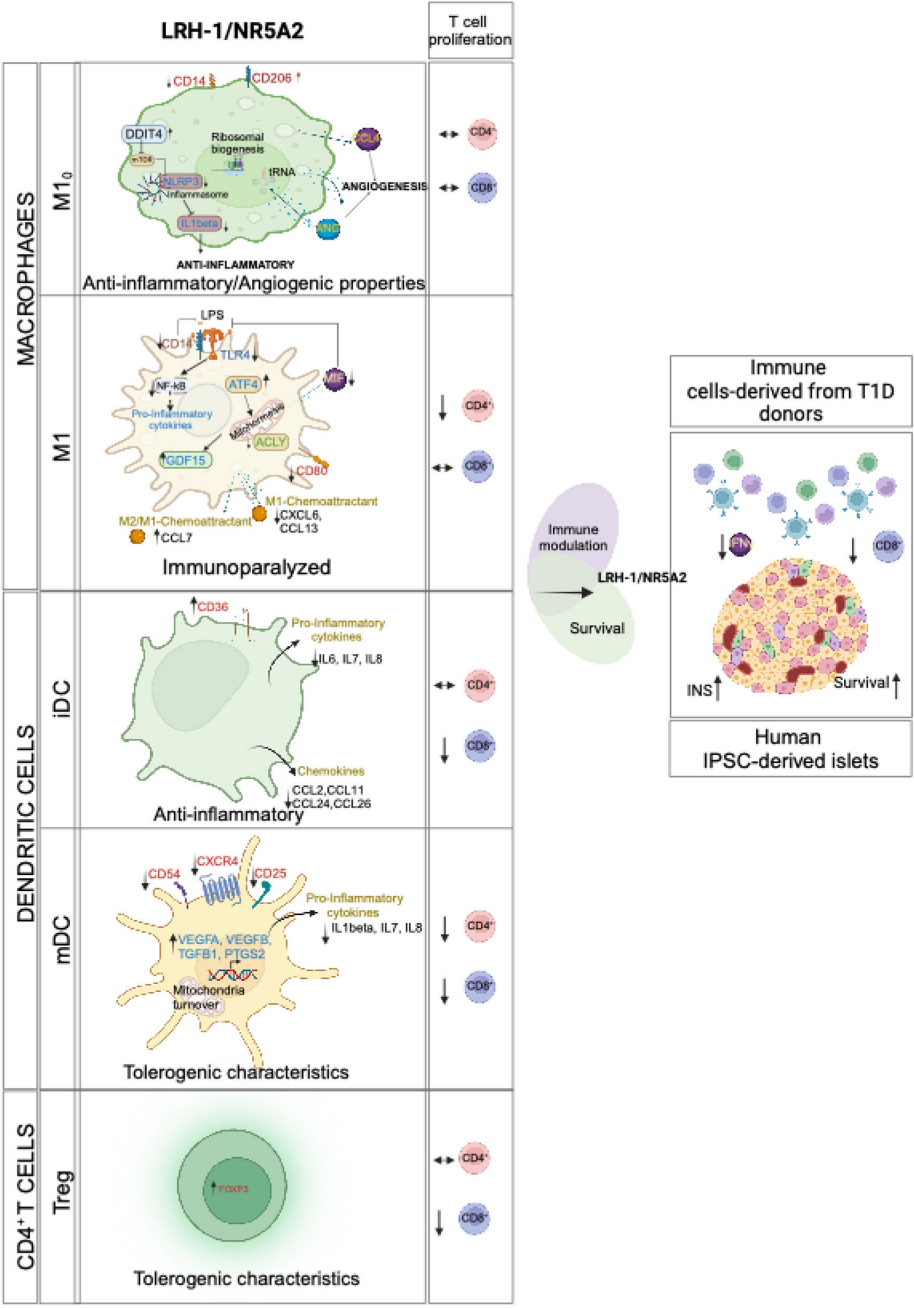
Cellular and molecular mechanism of action mediated by the pharmacological effect of LRH‐1/NR5A2 in human cells. Proposed model of genetic and immune cell‐tailored reprogramming induced by LRH‐1/NR5A2 activation and its impact on T‐effector cell proliferation.

Overall, our findings show that pharmacological activation of LRH‐1/NR5A2 drives the genetic and metabolic reprogramming of human MDMs, DCs and T‐cells. This reprogramming fosters an anti‐inflammatory and regenerative environment, promoting islet survival and function (Figure 8). Our study highlights the potential of LRH‐1/NR5A2 activation to *both* modulate immune responses and support β‐cell preservation, thus offering a promising and integrated strategy for T1D management. Future research should evaluate the immunomodulatory benefits of LRH‐1/NR5A2 agonists in newly diagnosed individuals and different age groups to optimise its clinical application and improve outcomes in T1D therapy.

## AUTHOR CONTRIBUTIONS

Nadia Cobo‐Vuilleumier, Roman González Prieto, Alejandro Martín‐Montalvo, Maria Isabel García Sánchez, Maria Asuncion Martínez‐Brocca, Marta Vives‐Pi, Decio L. Eizirik and Benoit R. Gauthier were involved in conceptualisation. Nadia Cobo‐Vuilleumier, Silvia Rodríguez‐Fernandez, Daniel Salas‐Lloret, Livia López‐Noriega, Beatriz Fernández‐Santos, Petra I. Lorenzo, Jaime M. Franco, Akaitz Dorronsoro, Eugenia Martin Vazquez, Raquel Araujo Legido, Christian C. Lachaud, Nila van Overbeek, Carmen Mateo‐Rodríguez, Lucia Hidalgo, Raul López‐Férnandez‐Sobrino, Alfred CO Vertegaal, Alejandro Martín‐Montalvo, Ana I. Arroba, Antonio Campos Caro, L. A. F., Sandra Marin‐Canas, Carmen Espejo Serrano, and Franz Martín contributed to the investigation, methodology and data acquisition. Nadia Cobo‐Vuilleumier, Silvia Rodríguez‐Fernandez, Livia López‐Noriega, Petra I. Lorenzo, Jaime M. Franco, Akaitz Dorronsoro, Christian C. Lachaud, Mireia Ramos‐Rodriguez, Lorenzo Piemonti, Marta Vives‐Pi, Roman González Prieto and Benoit R. Gauthier contributed to the formal analysis and data curation. Decio L. Eizirik, Rita Nano, Lorenzo Piemonti and Manuel Aguilar‐Diosdado provided resources. Nadia Cobo‐Vuilleumier and Benoit R. Gauthier wrote the manuscript. All authors were implicated in the review and editing of the manuscript. Project administration and funding acquisition was carried out by Benoit R. Gauthier, who is also the guarantor of this work and, as such, has full access to all the data in the study and takes responsibility for the integrity of the data and the accuracy of the data analysis.

## CONFLICT OF INTEREST STATEMENT

Two patents (WO 2011 144725 A2 and WO 2016 156531 A1) related to BL001 have been published of which B. R. G. and N. C. V. are inventors. M. V.‐P. holds a patent that relates to immunotherapy for T1D and is co‐founder and SEO of Ahead Therapeutics S.L., which aims at the clinical translation of immunotherapies for the treatment of autoimmune diseases. The other authors declare no competing interests related to the current study.

### ETHICS STATEMENT

The Clinical/Investigation Ethical Committees of the Hospital Germans Trias i Pujol and the University Hospital Virgen Macarena and Rocio approved all experiments with human islets and blood‐derived cells (PI‐19‐142, GAUTHIER‐201306722 and 2057‐N‐22) and have been conducted following the principles outlined in the Declaration of Helsinki for human research.

## Supporting information



Supporting Information

## Data Availability

All resources, reagents and raw data reported in this paper will be shared by the lead contact, Benoit R. Gauthier (benoit.gauthier@cabimer.es).The mass spectrometry proteomics data have been deposited to the ProteomeXchange Consortium via the PRIDE^104^ partner repository with the dataset identifier PXD045222. The RNAseq data have been deposited to the NCBI BioProject and can be retrieved with the project ID number PRJNA1017470.
