# Supplementary material for: LRH‐1/NR5A2 targets mitochondrial dynamics to reprogram type 1 diabetes macrophages and dendritic cells into an immune tolerance phenotype
Source: Clin Transl Med. 2024 Dec 19;14(12):e70134. doi: 10.1002/ctm2.70134 (PMC11659195; doi:10.1002/ctm2.70134)
Supplement: Supplementary file 1 — Supporting Information [file CTM2-14-e70134-s001.pdf]

Supporting Information

LRH-1/NR5A2 targets Mitochondrial Dynamics to Reprogram Type 1 Diabetes Macrophages and Dendritic Cells into an Immune Tolerance phenotype

Cobo-Vuilleumier *et al*.

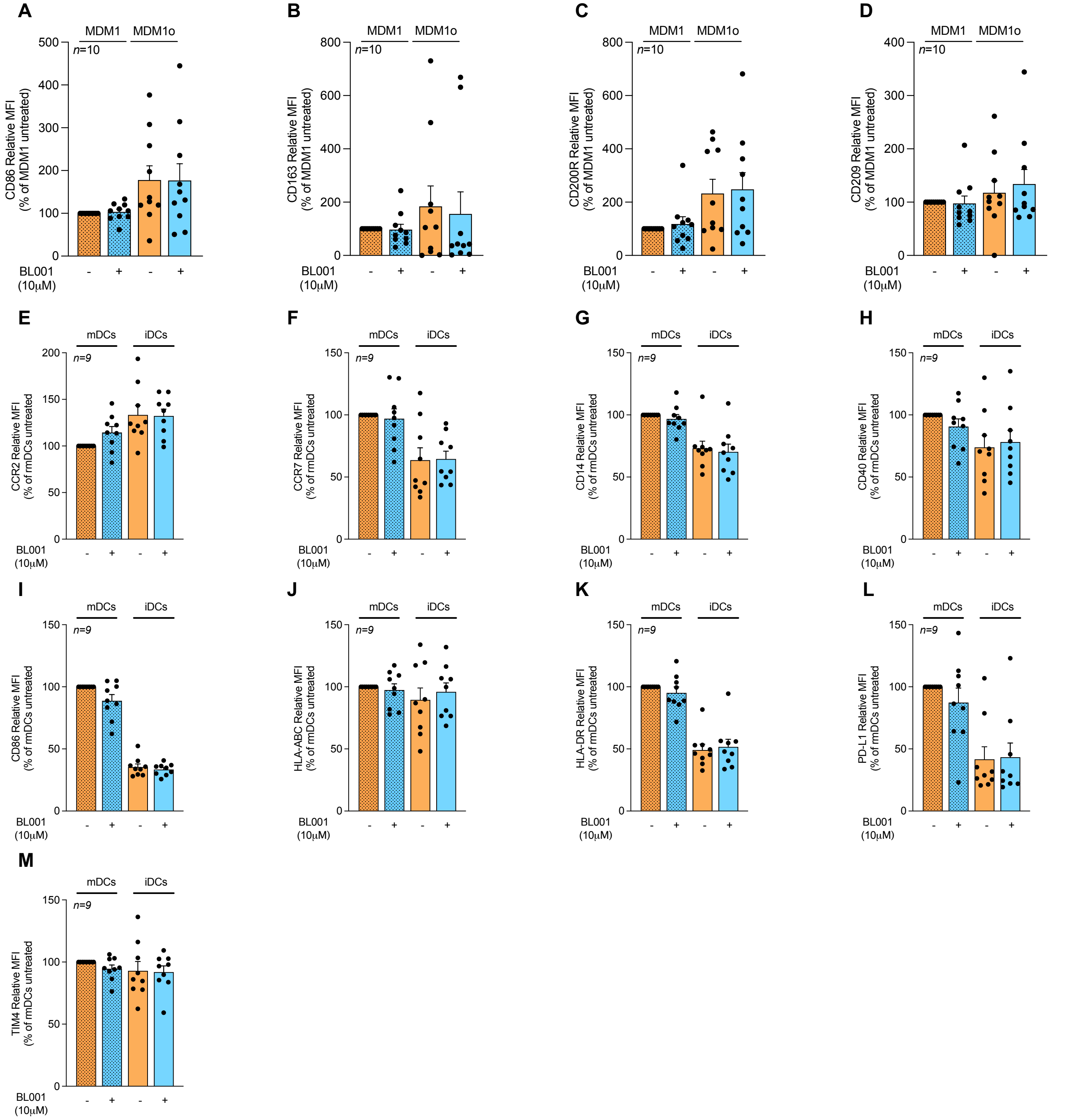

FIGURE S1. Cell surface markers of monocyte-derived macrophages (MDM) and dendritic cells (DCs) from individuals with T1D that were not altered by BL001 treatment. Monocytes were purified from individuals with T1D and derived into either resting or pro-inflammatory macrophage and dendritic cells (M1$_0$ or M1 and iDCs or mDCs) and treated with 10 uM BL001 for 48 hours and then 30 minutes prior to analysis. MDM cell surface markers (**A**) CD86, (**B**) CD163, (**C**) CD200R (D) CD209 and DC surface markers (**E**) CCR2, (**F**) CCR7, (**G**) CD14, (**H**) CD40, (**I**) CDCD86, (**J**) HLA-ABC, (**K**) HLA-DR, (**L**) PD-L1 and (**M**) TIM4 were then assessed by flow cytometry.

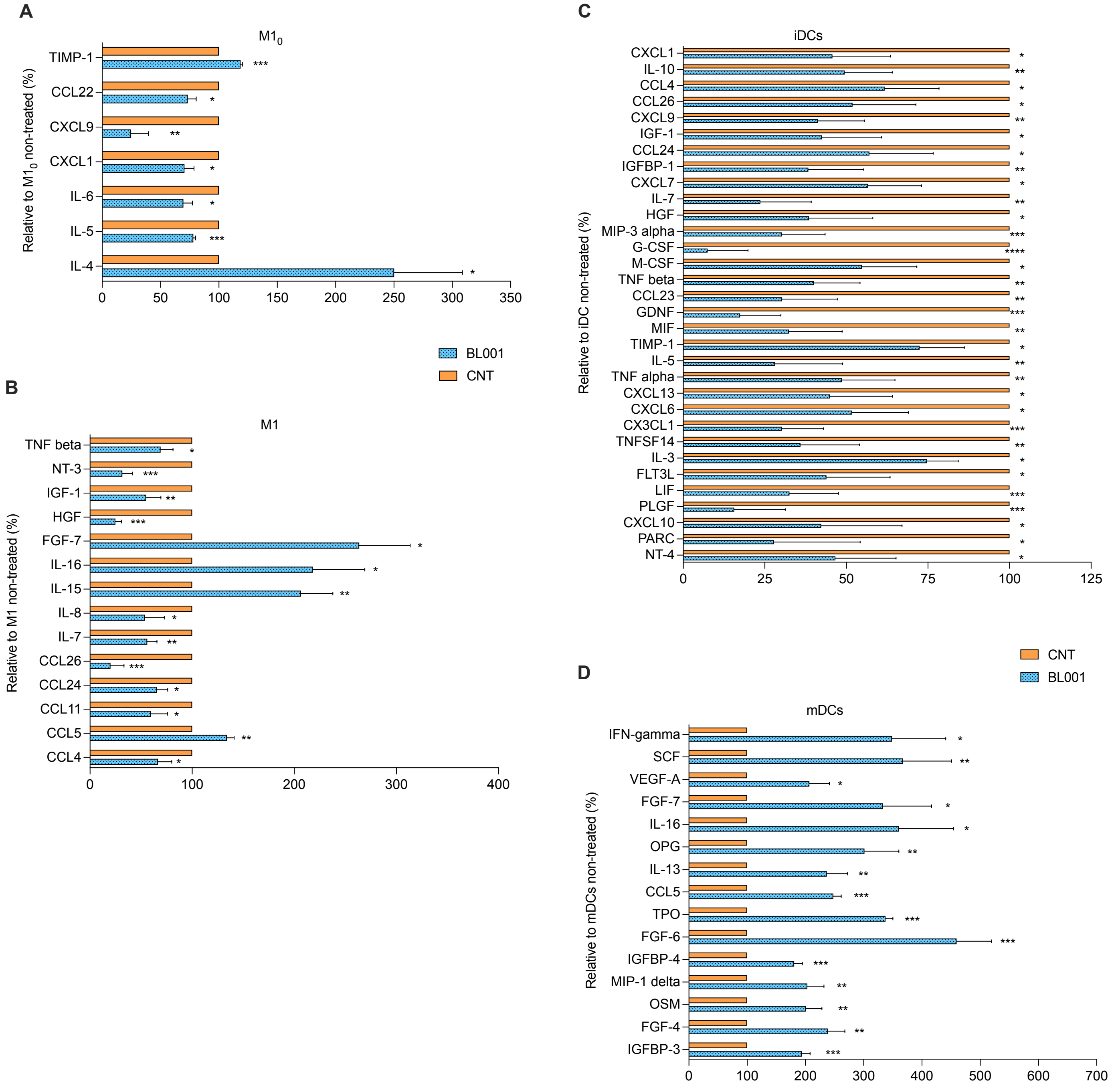

FIGURE S2. The cytokine secretion profile altered by BL001 in MDM and DCs from healthy donors differs from that observed in T1D individuals. Bar graphs depicting relative changes in cytokine secretion for healthy (**A**) M1$_0$, (**B**) M1, (**C**) iDCs and (**D**) mDCs, with treated values compared to their untreated counterpart. Only significantly altered cytokines are shown. Data are presented as percent changes compared to DMSO (non-treated) for each cytokine. Unpaired Student t-test * p<0.05, ** p<0.01, *** p<0.001, compared to DMSO (non-treated).

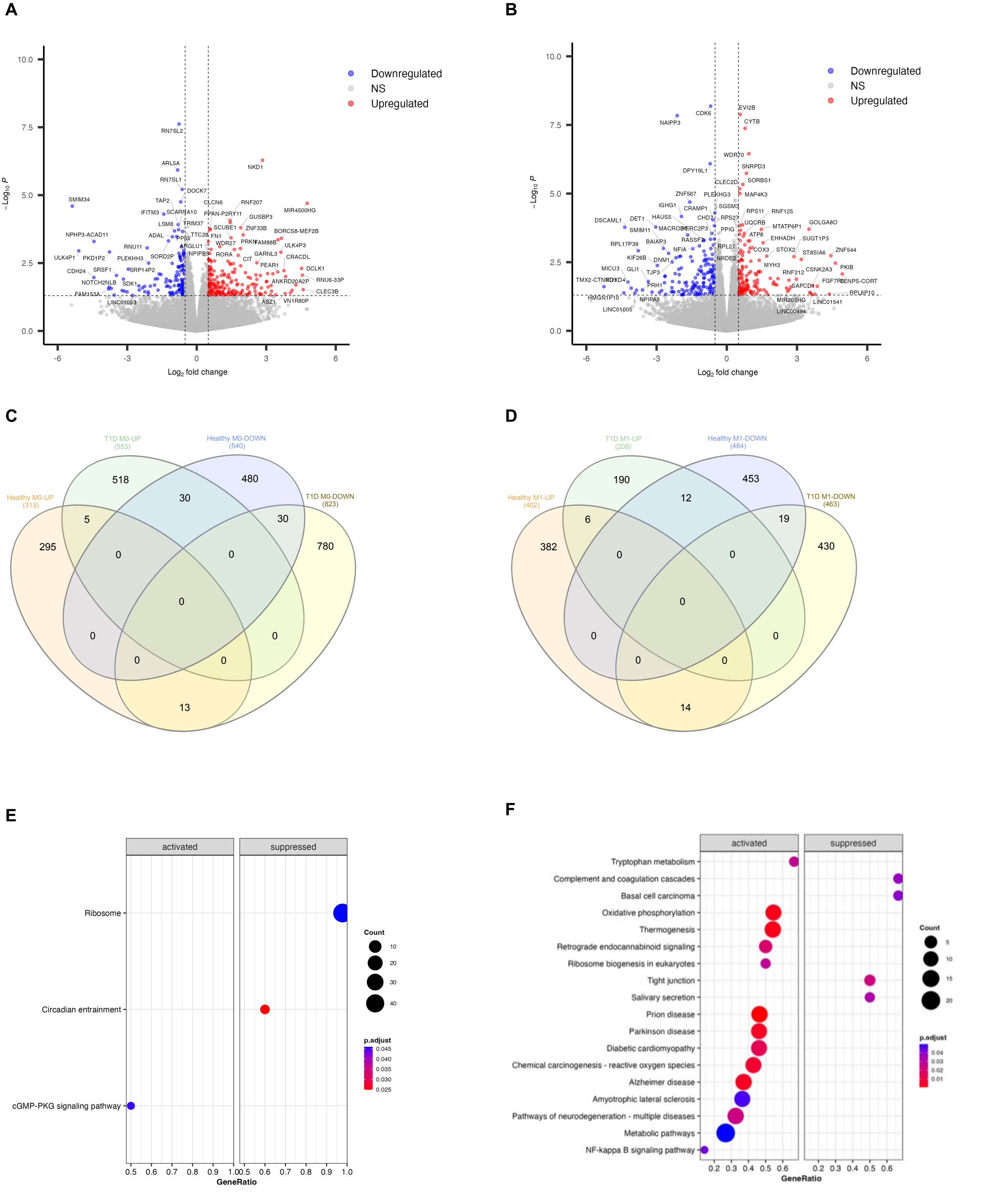

FIGURE S3. The genetic response of MDM to BL001 differs between healthy individuals and those with T1D. Volcano plot of differentially expressed genes (DEGs; *p*value <0.05 and $\text{Log}_2(\text{FC}) > 0.5$) in BL001-treated vs untreated (**A**) M1_0 and (**B**) M1 from healthy individuals (*n=4* independent donors). InteractiVenn diagram of up and-down regulated genes for healthy versus T1D (**C**) M1_0 and (**D**) M1. Dot plot of KEGG pathways enriched in BL001-treated (**E**) M1_0 and (**F**) M1 from healthy individuals (*n=4* independent donors).

KEGG TERM	Adjusted p value	GENES
Coronavirus disease - COVID-19	5.824370e-10	MX1,MX2,ISG15,OAS3,OAS2,OAS1,TLR7,STING1,EIF2AK2,FCGR2A,VWF,IRF9,STAT1,TLR8,PRKCA,FOS,IKBKE,NRP1,RPS15A,RPL3,RPL37A,RPL32,RPL9,RPS16,RPS13,RPS6,RPS3A,RPL30,RPL13A,RPL28,PRKCB,RPL23,RPS23,RPL15,RPS18,RPL13,RPL12,RPL18A,IRAK1,RPL17,RPLP0,MAPK13,CXCL8
Neutrophil extracellular trap formation	0.0000025864	H2AC14,H3C3,H2BC17,H3C2,H3C12,H3C7,H2BC3,H2AC12,H2BC14,H2BC10,H2BC11,H2AC17,H2AC7,H2AC16,H2BC6,TLR7,H2AX,H2AC21,FCGR3A,FCGR2A,VWF,HDAC9,TLR8,PRKCA,HDAC7,FPR3,H2BC12,ACTG1,MACROH2A1,PRKCB,ITGAL,SLC25A6,MAPK13,FPR1
Cell cycle	0.0000044095	CDK1,CDC25A,E2F2,CCNA2,CDC45,CDC20,CCNE2,PLK1,CDC6,E2F1,CCNB2,CDKN2C,BUB1,MCM7,CCNB1,MAD2L1,PKMYT1,CCND1,MYC,MCM3,PCNA,SKP2,MCM6,TFDP1,RB1,YWHAZ
Systemic lupus erythematosus	0.0002105552	H2AC14,H3C3,H2BC17,H3C2,H3C12,H3C7,H2BC3,H2AC12,H2BC14,H2BC10,H2BC11,H2AC17,H2AC7,H2AC16,H2BC6,CD80,HLADOA,H2AX,H2AC21,FCGR3A,FCGR2A,TRIM21,H2BC12,MACROH2A1
Ribosome	0.0076197549	RPS15A,RPL3,RPL37A,RPL32,RPL9,RPS16,RPS13,RPS6,RPS3A,RPL30,RPL13A,RPL28,RPL23,RPS23,RPL15,RPS18,RPL13,RPL12,RPL18A,RPL17,RPLP0
Alcoholism	0.0086872840	H2AC14,H3C3,H2BC17,H3C2,H3C12,MAOB,H3C7,H2BC3,H2AC12,H2BC14,H2BC10,H2BC11,H2AC17,H2AC7,H2AC16,GNG2,H2BC6,H2AX,H2AC21,PKIA,HDAC9,HDAC7,H2BC12,PRKACB,MACROH2A1,SLC29A1
Epstein-Barr virus infection	0.0124044321	E2F2,CCNA2,CCNE2,ISG15,E2F1,OAS3,OAS2,OAS1,HLADOA,IRF7,MAP2K6,EIF2AK2,CCND1,MYC,IRF9,ENTPD1,STAT1,CALR,RBPJ,SKP2,RB1,IKBKE,ITGAL,IRAK1,NCOR2,TNFAIP3,MAPK13
Viral carcinogenesis	0.0162088000	H2BC17,CDK1,H2BC3,CCNA2,CDC20,CCNE2,H2BC14,H2BC10,H2BC11,EGR3,H2BC6,IRF7,EIF2AK2,CCND1,HDAC9,IRF9,HDAC7,RBPJ,SKP2,H2BC12,PRKACB,RB1,PKM,YWHAZ,STAT5B,PXN,ATP6V0D2
Hepatitis B	0.0283354491	E2F2,CCNA2,CCNE2,E2F1,EGR3,BIRC5,NFATC2,IRF7,MAP2K6,MYC,STAT1,PRKCA,PCNA,FOS,RB1,IKBKE,YWHAZ,STAT5B,PRKCB,IRAK1,MAPK13,HSPG2,CXCL8

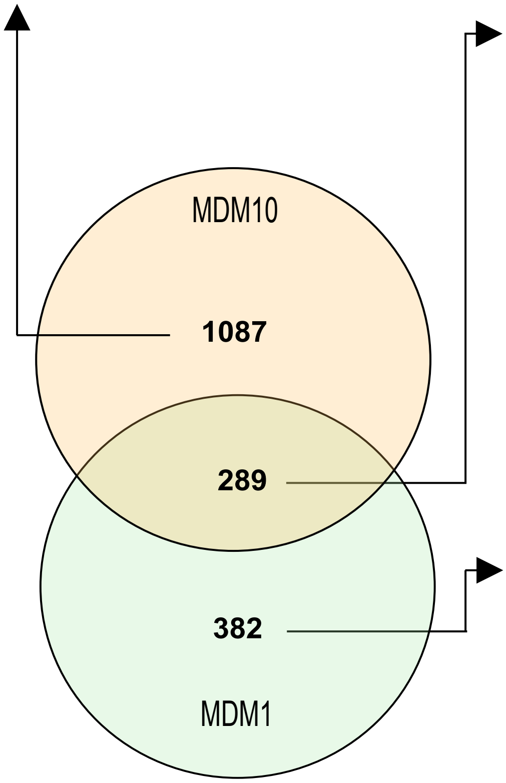

KEGG TERM	Adjusted p value	GENES
Hematopoietic cell lineage	0.0002254542	CD1E,CD1C,CD1B,ITGA4,IL1B,CSF1R,ANPEP,CD37,TFRC,FCER2,ITGB3
PPAR signaling pathway	0.0018393227	SCD,FADS2,HMGCS1,ACSL4,OLR1,ACSL3,PPARD,CYP27A1,PLIN2
Fatty acid metabolism	0.0018705126	SCD,FADS2,FADS1,ACSL4,ACSL3,ELOVL5,ACACA,ACADVL
Steroid biosynthesis	0.0031372665	DHCR24,SQLE,EBP,MSMO1,CYP51A1
Terpenoid backbone biosynthesis	0.0053098308	HMGCS1,HMGCR,IDI1,MVK,MVD
Ferroptosis	0.0126550104	MAP1LC3A,ACSL4,LPCAT3,ACSL3,SLC3A2,TFRC

KEGG TERM	Adjusted p value	GENES
NF-kappa B signaling pathway	0.0000023458	PLAU,CCL4L2,CCL4,CD14,CCL19,TRAF1,TLR4,MAP3K14,PTGS2,CD40,BIRC3,CFLAR,CYLD,NFKB1,RELB
TNF signaling pathway	0.0000066557	CSF1,MMP9,CCL5,IL15,NOD2,PIK3R3,TRAF1,MAP3K14,PTGS2,RPS6KA5,CASP7,BIRC3,MAP2K1,CFLAR,NFKB1
Toll-like receptor signaling pathway	0.0000133028	CCL4L2,CCL4,CD14,CXCL11,IL12B,CCL5,PIK3R3,TLR4,CCL3,CXCL9,TLR2,CD40,MAP2K1,NFKB1
Cytokine-cytokine receptor interaction	0.0001377890	CSF1,CCL4L2,CCL15,CCL4,CCL19,CXCL11,IL1A,TNFRSF9,IL12B,CCL5,IL2RA,ACVRL1,IL15,CCL3,CXCL9,EBI3,ACVR2A,CXCL16,CD40,IL13RA1,TNFRSF10B,IL27RA
Apoptosis	0.0017643701	CTSL,PIK3R3,TRAF1,MAP3K14,PMAIP1,CASP7,BIRC3,TNFRSF10B,BAK1,MAP2K1,CFLAR,CTSC,NFKB1
Chemokine signaling pathway	0.0129764758	CCL4L2,CCL15,CCL4,CCL19,CXCL11,CCL5,PIK3R3,CCL3,CXCL9,CXCL16,HCK,MAP2K1,NFKB1,ARRB1
Transcriptional misregulation in cancer	0.0154891784	MAF,MMP9,PLAU,CD14,TRAF1,FOXO1,SPINT1,CDKN1A,CD40,NFKBIZ,BIRC3,BAK1,ELK4,NFKB1

FIGURE S4: Distinct and common gene sets altered by BL001 in T1D MDM. A Venn diagram of the DEGs from BL001-treated M1_0 and M1 (pvalue < 0.05) was generated using InteractiVenn. Individual genes sets were then clustered in KEGG pathways using g:GOSt of g:Profiler.

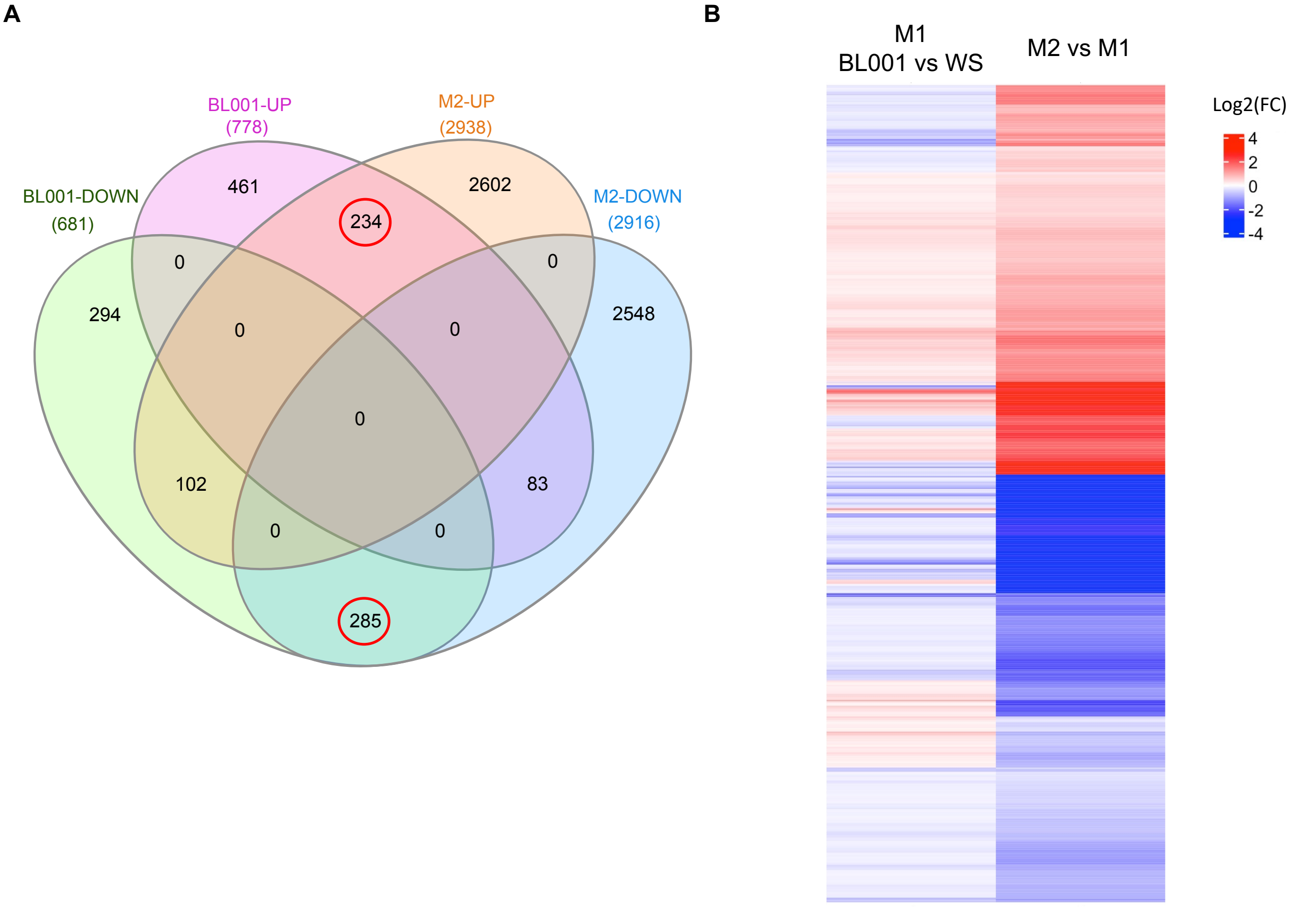

FIGURE S5. BL001 imposes a partial M2 gene signature to T1D M1. (**A**) An InteractiVenn diagram illustrating the overlap of up- and down-regulated genes in BL001-treated M1 macrophages compared to M2 and M1 macrophages, with the M2 versus M1 comparison extrapolated from public domain datasets. Red circles highlight the genes that are commonly up- or down-regulated in BL001-treated M1 macrophages and M2 macrophages. (**B**) Log2(FC) heatmap of DEGs (p-value < 0.05) comparing BL001-treated versus untreated (WS) M1 (1st column). The expression of these BL001-regulated genes was further analyzed in a comparison between M2 and M1 (2nd column), showing DEGs between M2 and M1 from healthy individuals (padj < 0.05)..

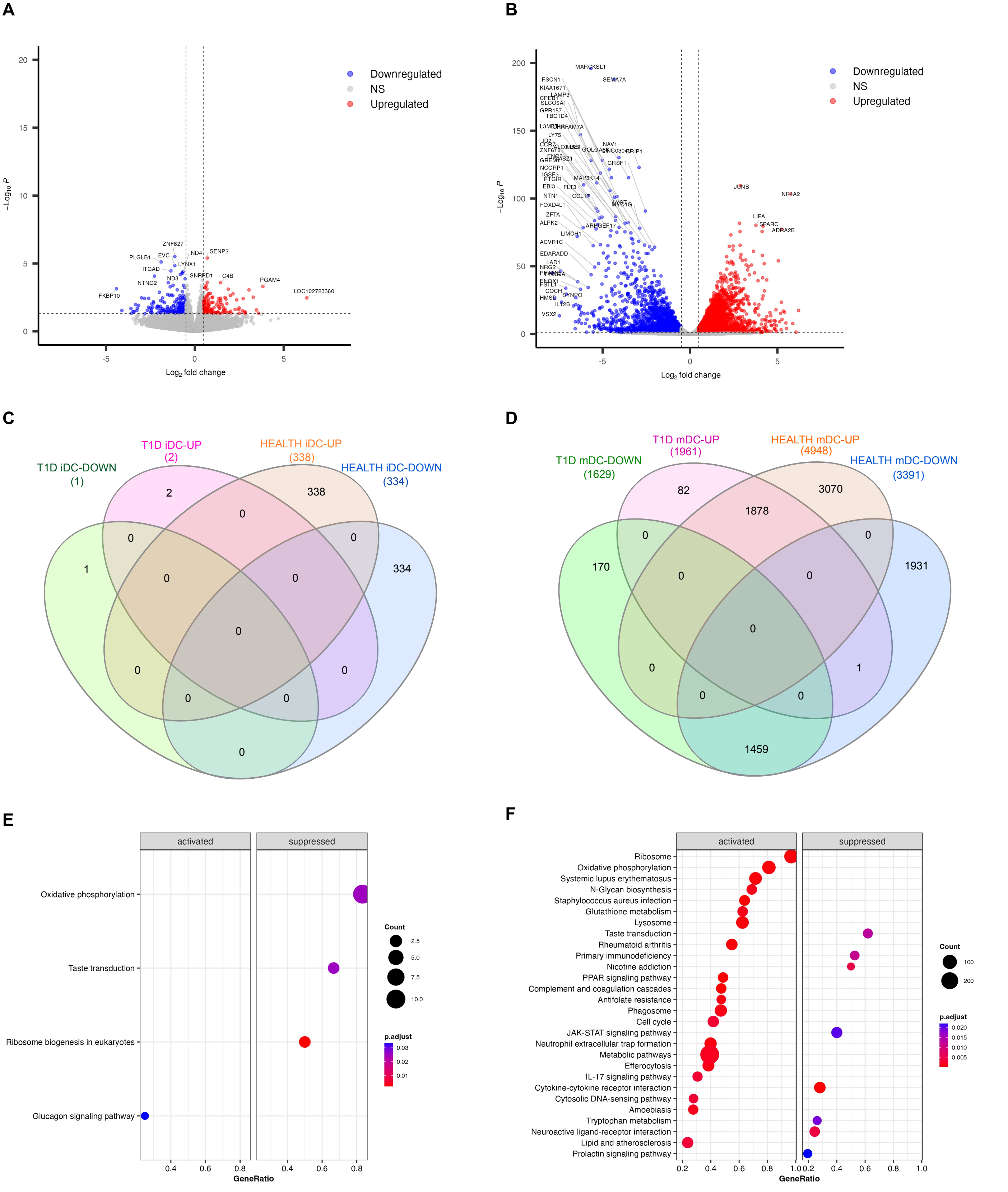

FIGURE S6. Similar genetic response of mDCs, but not iDCs, to BL001 between healthy individuals and those with T1D. Volcano plot of differentially expressed genes (DEGs; *p*value <0.05 and $\mathrm{Log}_2(\mathrm{FC}) > 0.5$) in BL001-treated vs untreated (**A**) idCs and (**B**) mDCs from healthy individuals (*n=5* independent donors). InteractiVenn diagram of up and-down regulated genes for healthy versus T1D (**C**) iDCs and (**D**) mDCs. Dot plot of KEGG pathways enriched in BL001-treated (**E**) iDCs and (**F**) mDCs from healthy individuals (*n=6* independent donors).

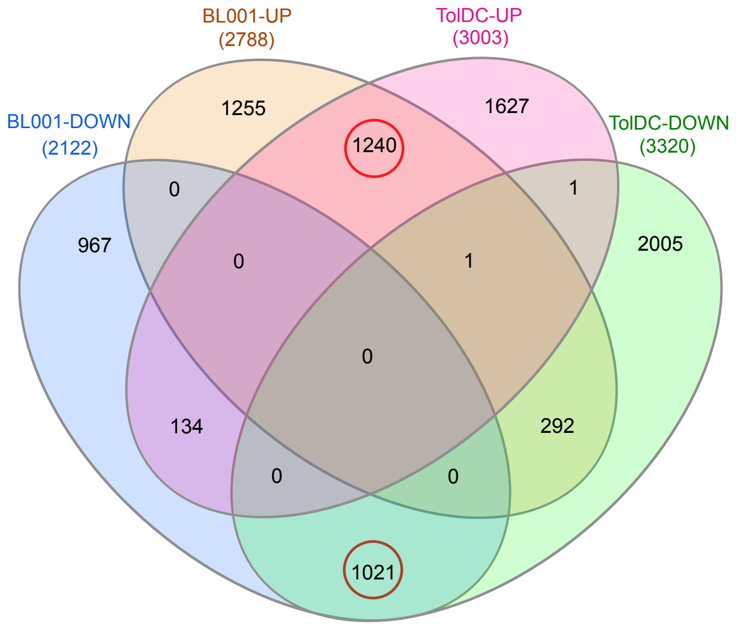

FIGURE S7. BL001 induces a tolerogenic phenotype to T1D mDCs. InteractiVenn diagram of up and-down regulated genes for BL001-treated mDCs (pvalue<0.05) versus TolDCs (padj<0.05). Red circles delineate the up and down-regulated genes common to both BL001-treated mDCs and TolDCs.

Table S1: Differentially expressed genes (*pvalue<0.05*) common to BL001-treated versus untreated M1 obtained from individuals with type 1 diabetes and M2 versus M1 obtained from public datasets.

Common up		Common down				
LRPAP1	ZBED3	ANKRD22	MAP1LC3A	ABCG1	TRANK1	DSE
DGLUCY	TNFSF14	CXCL9	GRAMD1A	OLR1	FAM135A	ARSD
GPC4	EOLA2-DT	UBD	MT1G	SYT11	ELAVL4	ARRB1
CSGALNACT2	ENOSF1	ACOD1	CD40	ACP3	PTPRJ	TSPAN14
UAP1L1	RPS6KA2	CCL19	ACVRL1	DENND5A	SH3BP1	ELK4
MSR1	CDC42EP3	ADORA2A	ICAM1	SNX20	RPS6KA5	CDC42SE1
GM2A	ADORA2B	CXCL10	SKIL	FILIP1L	FMNL3	ELOVL5
ASAH1	HAGHL	CCL15	LMNB1	STARD4	NR3C1	
PACS2	LGALS3	CXCL11	DKK2	CD300E	PPP1R16B	
COL6A1	DENND4C	CD38	RASGRP1	RAPGEF2	SFT2D2	
USF2	THRA	IL2RA	SERPINA1	SERPINB9	AKR1B1	
NCS1	ALDH1L2	TNFAIP6	SLC39A8	DAPP1	ECE1	
GNPDA1	TM4SF19	GBP1P1	MT1E	HELZ2	CXCL16	
PGM3	SCARB1	IL12B	CCL3	LST1	CSRNP1	
GASK1B	SLC17A9	EBI3	PTGER4	H2BC5	SLC35C2	
CYP27A1	RAB3IL1	GBP4	CCL4	ZMYND15	SLC43A2	
ZMIZ1	CD22	TNIP3	NHS	CFLAR	ATP2C1	
AMDHD2	PHGDH	NTN1	EREG	TP53INP2	PRRG4	
KLF10	TMC8	GCH1	NFKBIZ	ADM	IDS	
MGAT1	MCOLN3	CCL1	PTGIR	FSCN1	CD14	
LINC00847	ACP5	CCL5	S100A8	SLC8A1	PPARD	
VEGFB	LIPA	HS3ST3B1	GPR132	ALDH2	RASSF4	
PLIN2	SLC26A11	PDE4B	PNRC1	STK26	SGMS1	
NPL	LY9	CALHM6	C3AR1	C21orf91	AHCYL2	
PHYH	RTN4R	OSM	PLEKHG1	SIGLEC10	F3	
NCLN	APBA1	GBP1	MGAM	MXD1	SERPINB8	
RNF130	AHRR	SYNPO2	LRRK2	DUSP5	CMTM6	
MICAL1	CR1	MT1M	SLC9A7P1	UBE2D1	KPNB1	
ACAA2	SHB	APOL3	RAB29	HCK	CRIM1	
VAT1	MAF	LAMP3	MARCKSL1	CD82	SLCO3A1	
GATD1	DNM1	NCF1B	ASAP2	CYLD	ARSB	
HEXB	FCER2	CLEC4E	TMEM229B	KIAA0040	ITPRIPL2	
CD300A		ANKRD33B	CSF2RB	MARCKS	SLC44A1	
ERMP1		PTGS2	ASPHD2	PIK3R3	ST3GAL2	
RRAGD		CLEC6A	H2BC4	NFAM1	CA11	
SOX13		NCF1C	PMAIP1	ANTXR2	ITPRIP	
ITGB3		C15orf48	EHD1	PLA2G4A	SMPDL3A	
AHSA2P		TNFAIP2	DRAM1	TNFAIP8	TMED7-TICAM2	
SBF2-AS1		MT1H	TNIP1	LMTK2	LDLR	
PHACTR1		CD69	SLC6A12	FCAR	MRTFA	
SLC4A8		IL1B	SEMA4A	SLC16A3	SRD5A1	
KIAA0930		SYNPO	TRAFD1	EHBP1L1	RTN1	
ZMIZ1-AS1		CCL3L1	NFKB1	TRAF1	FARP2	
STRBP		SLC2A6	LYSMD2	IL10RA	PTPRC	
ZFYVE28		FPR2	RELB	CBX6	ARL6IP5	
SLC28A3		CRISPLD2	NEMP1	TAGLN2	RDH11	
CSTB		TNFRSF9	NFE2L3	ACSL4	SLC36A4	
KCTD7		IL1A	MT1X	NLRP3	GCLM	
RAB42		HSH2D	NOD2	CLIC4	NFAT5	
OSBPL1A		CCL4L2	APOBEC3G	CLINT1	SQLE	
GPNMB		MT1F	CLEC5A	TLR2	LPGAT1	
ST14		ZC3H12A	CGAS	FLOT2	PDLIM7	
C1orf162		HIVEP2	NUAK2	RASSF5	INSIG1	
ITPKB		ADAM19	NCOA4	CDC42SE2	VCP	
PDE3B		IL15	SINHCAF	SLC30A1	SC5D	
KCNC4		RIPOR2	SORBS1	NIBAN1	BAK1	
CD109		HBEGF	KCNJ2	IL13RA1	CD46	
MATK		BIRC3	SRGN	ABCA1	CASP7	

Table S2: Proteins which are significantly (*p*value<*0.05*) decreased in BL001-treated $M1_0$ obtained from individuals with type 1 diabetes.

Protein	-Log(p)	Log2(FC)
BST2	1,66	-1,55
SLC25A1	1,50	-1,32
SLC35F6	1,35	-1,21
VDAC1	2,22	-1,13
ALCAM	1,51	-1,11
UNC93B1	1,88	-1,11
AASDHPPT	1,75	-1,09
MIA3	1,36	-1,04
UNC13D	2,14	-0,96
LSM5	2,01	-0,92
SLC43A3	1,69	-0,90
DNAJB6;DNAJB7	1,64	-0,87
VDAC3	1,31	-0,86
TM9SF2	1,38	-0,84
S100A10	1,47	-0,83
LMNA	1,59	-0,81
NDUFV1	1,33	-0,79
S100A11	1,82	-0,77
TGOLN2	1,51	-0,77
SNRPB2	1,36	-0,77
TM9SF3	1,42	-0,77
COX6B1	1,31	-0,72
LSS	1,71	-0,67
NDUFS1	1,46	-0,67
RBM3	1,36	-0,65
RPN2	1,47	-0,64
FASN	3,03	-0,62
GIMAP8	2,53	-0,61
CD86	1,41	-0,60
ATP1B3	1,98	-0,60
BCAP29	1,41	-0,57
ZYX	2,64	-0,52
ATP1A1	2,88	-0,51
ATP2A3	2,44	-0,51
PSMA2	1,32	-0,51
LRRC8C	1,95	-0,49
MRC1	2,29	-0,48
UQCRH	1,73	-0,48
HNRNPA1;HNRNPA1L2	2,46	-0,48
CORO7;CORO7-PAM16	2,79	-0,47
PPP1CC	1,39	-0,47
IFI35	1,40	-0,47
FTH1	1,74	-0,47
ARHGEF6	1,45	-0,47
VDAC2	1,80	-0,45
DNAJA1	2,32	-0,45
UQCRC2	2,02	-0,45

Protein	-Log(p)	Log2(FC)
SIGLEC9	1,49	-0,45
EHBP1L1	1,63	-0,44
SF3A2	1,47	-0,44
ERP44	1,45	-0,41
UQCRFS1;UQCRFS1P1	1,89	-0,40
MAGED2	1,31	-0,40
CD97	2,69	-0,38
RPLP2	1,64	-0,37
HADHB	1,58	-0,36
GMFG	2,65	-0,36
NDUFB3	1,38	-0,36
GBF1	1,38	-0,35
EVL	1,40	-0,35
FDPS	1,65	-0,34
MED15	1,44	-0,34
EVI2B	2,20	-0,34
LCP1	1,43	-0,33
VMP1	1,48	-0,33
APBB1IP	1,44	-0,32
PPIA	1,59	-0,30
RPRC1;MAP7D1	1,39	-0,30
RNF213	2,12	-0,30
VASP	1,44	-0,29
HNRNPA3	1,32	-0,29
PRDX5	1,32	-0,29
TES	1,38	-0,25
SH3BP1	1,39	-0,24
APRT	1,64	-0,23
DNAJB11	1,72	-0,23
RBMX	1,51	-0,23
COX7A2	1,31	-0,23
PTPN12	1,92	-0,22
SAMHD1	1,38	-0,21
PNPLA6	1,38	-0,20
ERH	1,45	-0,19
HCK	2,33	-0,19
S100A9	1,32	-0,18
PICALM	1,89	-0,18
TUBA1B	2,12	-0,17
HNRNPD	1,49	-0,11
DNAJC3	1,84	-0,11
UBAP2L	1,30	-0,11
Sep-02	1,33	-0,10
PGAM1	2,67	-0,09
TLN1	1,69	-0,09

Table S3: Proteins which are significantly (*pvalue<0.05*) increased in BL001-treated $M1_0$ obtained from individuals with type 1 diabetes.

Protein	-Log(p)	Log2(FC)
CCT4	1,50	0,08
DYNC1H1	1,46	0,10
RPL23A	1,83	0,10
UBE2N;UBE2NL	1,40	0,12
CCT5	1,40	0,12
RTCB	1,31	0,12
PLEC	1,45	0,13
FERMT3	1,74	0,14
CCDC88A	1,95	0,15
SND1	2,48	0,16
PDCD5	1,31	0,18
BCAP31	1,58	0,18
EPRS	1,91	0,18
NSF	1,86	0,18
STIP1	2,05	0,18
CAPG	2,15	0,18
RARS	2,12	0,19
YARS	1,68	0,20
NME1-NME2;NME2	2,75	0,20
RUFY1	1,74	0,20
ALYREF	1,76	0,20
EIF4A1	1,46	0,21
EIF4G2	1,66	0,21
AHCY	3,15	0,21
SNX8	1,45	0,22
PRRC1	2,99	0,24
PSMA6	1,44	0,24
KYNU	1,31	0,26
AP2M1	2,20	0,26
AGPS	1,33	0,26
RPS14	2,29	0,27
LRPPRC	2,86	0,27
ATP6V1G1	2,21	0,27
TBXAS1	1,67	0,28
PHB	1,37	0,28
PDS5B	1,32	0,28
RPN1	1,80	0,28
FGR	1,73	0,29
ATP6V1D	1,69	0,30
PRKCB	1,33	0,30
UFL1	1,52	0,30
COPA	1,44	0,30
SULT1A4;SULT1A3	1,42	0,30
TALDO1	1,63	0,30
MOB1A	1,50	0,32
SACM1L	2,20	0,33
SGPL1	1,37	0,34
CES1	1,46	0,34
CHMP4B	1,66	0,34
TARDBP;TDP43	1,56	0,37
GRPEL1	1,51	0,37
EMC2	2,79	0,37
ALDH3A2	2,66	0,37
TNS3	1,69	0,37
AARS	1,57	0,38
RAB18	1,63	0,39
EIF2S3;EIF2S3L	2,42	0,40
GLS	1,91	0,40
ARPC5	1,59	0,41
OTUB1	1,51	0,41
DARS	1,62	0,43
HIST1H1E	1,89	0,43
ADD1	1,39	0,44
DHRS4	1,42	0,44
CLPB	2,72	0,46
LRRC47	1,48	0,47
PSAP	2,58	0,50
IRF5	1,95	0,50
CPPED1	1,67	0,53
VAT1	3,07	0,55
FLNB	1,41	0,55
ATXN3	1,31	0,56
COL4A3BP	1,47	0,57
PIK3R1	1,57	0,59
IPO9	1,64	0,62
CTSD	1,79	0,66
PML	1,66	0,68
FCHO2	2,33	0,70
TBC1D2	1,41	0,70
SHMT2	1,36	0,75
PLOD3	1,35	0,85
PC	1,33	0,86
OAT	2,68	0,92
CALCOCO1	1,89	0,92
SQSTM1	2,29	1,12
CHCHD2;CHCHD2P9	2,55	1,13
CD40	1,54	1,15
GM2A	2,47	1,42
TFRC	1,68	1,79

Table S4: Proteins which are significantly (*pvalue<0.05*) decreased in BL001-treated M1 obtained from individuals with type 1 diabetes.

Protein	-Log p	Log2(FC)
CD14	2,15	-2,28
FAM175B	1,33	-1,18
CD163	1,42	-1,12
CYCS	1,62	-1,10
ACO1	2,20	-0,96
LYPLA2	1,54	-0,93
S100A10	1,68	-0,92
UNC13D	1,58	-0,88
RPN2	1,68	-0,85
SERPINH1	2,59	-0,84
ADPGK	1,50	-0,75
PSMA2	1,65	-0,68
PSMB10	1,41	-0,65
MT-CO3	2,24	-0,60
SIRPA;SIRPB1	2,24	-0,58
GNB2	1,63	-0,58
EHD1	2,22	-0,57
U2AF2	1,62	-0,57
PTGR1	1,38	-0,56
TAPBP	2,96	-0,56
PAFAH1B2	1,45	-0,56
PGLS	1,77	-0,53
RPS2	1,34	-0,51
TIMM50	2,25	-0,51
HIP1	1,82	-0,50
ACTL6A	1,36	-0,49
NDUFA9	2,38	-0,49
SEC11A	1,37	-0,48
ERAP1	2,13	-0,46
ACSL4	1,76	-0,45
QARS	2,25	-0,44
CRYZ	1,44	-0,42
THEMIS2	1,67	-0,40
HNMT	1,82	-0,40
CTSZ	1,54	-0,39
UBE2N;UBE2NL	2,12	-0,38
GBF1	1,61	-0,37
VPS13C	1,44	-0,36
DCTN1;DKFZp686E0752	1,67	-0,34
VCL	1,55	-0,34
ATP2A3	1,46	-0,33
COTL1	1,35	-0,33
TMEM43	1,41	-0,33
CASP1	1,49	-0,33
MTAP	1,51	-0,32
TMEM173	1,31	-0,32
TMCO1	1,62	-0,32
PPA1	1,92	-0,29
PSMA4	1,71	-0,28
RAP1B	1,61	-0,26
COPG2	1,31	-0,25
MAPRE1	1,55	-0,24
CCDC124	1,44	-0,23
HPRT1	1,57	-0,19
RNF213	1,58	-0,18
PFN1	1,35	-0,18
PSMD7	2,09	-0,18
ACLY	1,32	-0,18
EEF2	1,53	-0,18
AP2A1	1,71	-0,17
ATP5F1	1,39	-0,17
FMNL1	1,53	-0,16
PRDX6	1,97	-0,14
ANXA6	2,48	-0,12
SARS	1,37	-0,10
ADSS	1,75	-0,05

Table S5: Proteins which are significantly (*pvalue<0.05*) increased in BL001-treated M1 obtained from individuals with type 1 diabetes.

Protein	-Log p	Log2(FC)
RAB14	1,36	0,08
NUCB1	1,55	0,08
ANXA11	1,60	0,09
PHB	2,06	0,10
MVP	1,60	0,13
XRCC6	1,51	0,14
LRRC47	1,70	0,17
ACADVL	1,80	0,18
CCDC88A	1,34	0,20
PNN	1,30	0,21
EIF6	1,43	0,21
RPS18	2,29	0,24
ECHS1	2,16	0,24
P4HB	2,86	0,25
CLIP1	1,35	0,25
NCLN	1,59	0,26
WDR61	2,47	0,28
RPL7A	1,34	0,28
GRPEL1	1,37	0,29
TCEA1	1,79	0,29
HNRNPA3	1,33	0,30
DDX17	1,37	0,30
CLASP2	1,94	0,30
PPIF	1,45	0,30
NUDC	1,82	0,31
EDC4	2,40	0,33
KHSRP	2,18	0,33
ST13	2,17	0,33
HCFC1	1,61	0,33
SMARCE1	1,45	0,34
FBL	1,88	0,37
XRN2	1,38	0,38
PPP1CC	1,81	0,39
NUP93	1,92	0,40
VAMP8	1,72	0,40
GIMAP1	2,09	0,41
SLC4A1AP	3,37	0,42
NRP1	1,54	0,43
POLR2A	1,91	0,46
SART1	1,43	0,53
NOMO1	1,90	0,57
NUP62	1,63	0,58
TDP43	1,79	0,60
TBL1XR1	1,34	0,60
GOLGA2	1,73	0,66
FUS	2,81	0,71
SOAT1	1,36	0,72
RPL36AL	1,80	0,76
RPL8	1,33	0,80
GOLGB1	1,64	0,90
HIST1H1E	1,50	0,93
EIF4A1	1,73	2,32
SPTBN1	1,72	2,71

Table S6: Proteins which are significantly (*p<0.05*) decreased in BL001-treated mDCs obtained from individuals with type 1 diabetes.

Protein	-Log p	Log2(FC)
KRT74	1,62	-3,10
PDCD2	1,38	-1,39
ACTG2	1,34	-1,03
PIK3CB	1,76	-1,01
KRT84	2,20	-0,92
ITFG1	1,33	-0,85
COL9A3	1,32	-0,78
IFIT3	1,73	-0,73
CD3E	1,91	-0,60
PRNP	1,93	-0,60
TMEM209	1,38	-0,57
NOL3	1,58	-0,56
TPST2	1,39	-0,55
RFC1	1,64	-0,54
HLA-A;HLA-H	2,14	-0,49
CDC42SE2	1,36	-0,45
MYH1;MYH13;MYH4;MYH8	1,57	-0,42
UQCRH	1,63	-0,35
CBX1;CBX3	1,42	-0,35
GATAD2B	1,41	-0,35
STT3A	1,46	-0,34
ASDURF	1,59	-0,30
GGCT	1,39	-0,29
DAZAP1	3,19	-0,27
EIF5A;EIF5A2	1,47	-0,26
STX11	1,93	-0,23
RTN3	1,66	-0,22
PEA15	1,31	-0,22
COMT	1,30	-0,21
TUBB;TUBB2A;TUBB4B	1,36	-0,21
UBL4A	1,87	-0,21
BAX	1,32	-0,20
ERBIN	1,51	-0,20
TUBB4A;TUBB4B	2,05	-0,19
PPP4C	1,68	-0,18
RPL9	1,38	-0,18
SEC23A	1,51	-0,18
APEX1	1,52	-0,16
FAM98B	1,38	-0,15
THEMIS2	1,81	-0,12
MATR3	1,50	-0,10
PRDX6	1,60	-0,10
ABCD1	1,76	-0,08
MACROH2A1	1,48	-0,07

Table S7: Proteins which are significantly (*p<0.05*) increased in BL001-treated mDCs obtained from individuals with type 1 diabetes.

Protein	-Log p	Log2(FC)
GNPDA1	1,37	0,05
APPL1	1,65	0,07
CORO1B	1,42	0,07
DNAJC1	1,76	0,08
ARFGEF1;ARFGEF2	1,31	0,08
PPIB	1,41	0,08
SRP54	1,30	0,08
VPS4B	1,61	0,08
GLUD1	1,65	0,09
RAN	1,93	0,09
SARS2	1,97	0,09
BUB3	2,12	0,09
PSMD11	1,37	0,10
FARSB	1,43	0,10
HS1BP3	1,81	0,10
CRYZ	4,01	0,10
ELAVL1	1,50	0,10
HSP90B1	1,93	0,10
MRPL12	1,46	0,10
STK24;STK26	1,64	0,11
ATP6V0D1	1,58	0,11
VPS16	1,40	0,11
ACADVL	1,68	0,11
RTRAF	1,30	0,11
SAE1	1,31	0,11
CTBP1	1,71	0,11
FMR1;FXR1;FXR2	2,69	0,11
DLAT	1,69	0,12
RPS3	1,48	0,12
GANAB	2,75	0,12
SNX1	1,43	0,12
RPL10A	1,31	0,12
CCT6A;CCT6B	1,82	0,12
RAB1A;RAB1B	1,99	0,12
MRE11	1,41	0,13
SNU13	1,81	0,13
TRAPPC3	1,52	0,14
ELMO1;ELMO2	1,50	0,14
ADSL	1,63	0,14
ATP5F1A	1,60	0,14
ATP5PO	1,78	0,14
MRPL13	1,52	0,14
MBNL1	1,50	0,14
NIT1	1,42	0,14
LILRA1;LILRB1	1,32	0,15
COMMD7	1,34	0,15
DCPS	2,19	0,15
TAOK1;TAOK3	2,10	0,15
POFUT1	1,72	0,15
HEATR3	1,34	0,15
P4HA1	2,08	0,15
TRAPPC11	1,58	0,16
ATP6V1G1	2,27	0,16
ERP29	1,40	0,16
ACOT1;ACOT2	1,34	0,16
PIK3CG	1,36	0,16
WDR81	1,45	0,16
ACADSB	1,67	0,17
EXOC8	1,63	0,18
RPS4X;RPS4Y1;RPS4Y2	1,49	0,18
POLR2B	1,39	0,18
ELMO1	2,17	0,18
MESD	1,45	0,18
RPL15	1,36	0,18
ARL3	2,34	0,19
EIF1;EIF1B	1,43	0,19
GSDMD	1,30	0,19
ALDH18A1	1,38	0,19
TM9SF3	1,65	0,20
MCCC2	1,69	0,21
RABGAP1	2,52	0,21
OPTN	1,51	0,22
AIMP2	2,61	0,23
AGAP3	1,37	0,24
SRP9	2,22	0,24
ARSB	1,52	0,25
NCOR2	1,40	0,25
PWP1	3,12	0,25
NOP10	1,70	0,25

Protein	-Log p	Log2(FC)
CIAO1	1,90	0,26
GMFB;GMFG	1,97	0,26
OXSR1	1,34	0,26
RMC1	1,34	0,27
TAF4	2,00	0,27
MMAB	1,58	0,27
MRPL11	1,91	0,27
CTNNA1;CTNNA3	1,39	0,27
RPL11	1,93	0,30
BUD31	2,64	0,31
RRM2B	1,65	0,31
AKT2	2,08	0,31
AVEN	1,33	0,32
MRPL23	1,48	0,33
CBR4	1,67	0,34
UBASH3A	2,10	0,34
SEPTIN3	1,70	0,36
EARS2	2,70	0,37
COX17	1,35	0,39
IGHG2;IGHG4	1,43	0,39
MRPL1	1,31	0,40
EED	2,27	0,40
TMEM245	1,76	0,40
MAOB	1,33	0,40
TANGO6	1,46	0,41
PTTG1IP	1,43	0,41
FYN;SRC;YES1	1,67	0,41
ALKBH7	1,75	0,42
SP1	1,77	0,47
SNX1;SNX2	1,80	0,47
RILP	1,44	0,48
UQCC2	1,35	0,50
CSNK1E	1,87	0,54
LCK	1,50	0,54
ATF6	1,54	0,55
CASTOR1;CASTOR2	1,39	0,56
ERVK-6;HERVK_113	2,18	0,56
EEPD1	1,63	0,58
UXS1	1,36	0,58
ATAD2B	1,39	0,58
PUM3	1,42	0,59
BZW1;BZW2	1,40	0,62
AIDA	1,46	0,64
KNG1	1,32	0,64
OXSM	1,36	0,64
NT5C3A	1,33	0,67
ISY1	1,96	0,68
NELFCD	1,60	0,73
VAMP2	1,38	0,74
WDR74	1,61	0,75
GNPDA2	1,64	0,55
APPL2	1,64	0,56
CORO1B	1,64	0,56
DNAJC2	1,64	0,57
ARFGEF1;ARFGEF3	1,64	0,57
PPIB	1,63	0,57
SRP55	1,63	0,58
VPS4B	1,63	0,58
GLUD2	1,63	0,59
RAN	1,63	0,59
SARS3	1,63	0,60
BUB4	1,63	0,60
PSMD12	1,63	0,61
FARSB	1,63	0,61
HS1BP4	1,63	0,61
CRYZ	1,63	0,62
ELAVL2	1,63	0,62
HSP90B2	1,63	0,63
MRPL13	1,63	0,63
STK24;STK27	1,62	0,64
ATP6V0D2	1,62	0,64
VPS17	1,62	0,65
ACADVL	1,62	0,65
RTRAF	1,62	0,66
SAE2	1,62	0,66
CTBP2	1,62	0,66
FMR1;FXR1;FXR3	1,62	0,67
DLAT	1,62	0,67

Table S8: GO enrichment terms associated to mitochondria for up-regulated proteins in BL001-treated mDCs as compared to untreated mDCs.

GO Biological processes	Proteins
mitochondrial translation	SARS2/MRPL12/MRPL13/MRPL11/MRPL23/EARS2/MRPL1/UQCC2/MRPL3
mitochondrial gene expression	SARS2/MRPL12/MRPL13/MRPL11/MRPL23/EARS2/MRPL1/UQCC2/MRPL3
mitochondrial translational elongation	MRPL12/MRPL13/MRPL11/MRPL23/MRPL1/MRPL3
mitochondrial translational termination	MRPL12/MRPL13/MRPL11/MRPL23/MRPL1/MRPL3
mitochondrial RNA metabolic process	SARS2/MRPL12/EARS2
mitochondrial ATP synthesis coupled proton transport	ATP5F1A/ATP5PO
inner mitochondrial membrane organization	ATP5F1A/ATP5PO
mitochondrial respiratory chain complex III assembly	UQCC2
positive regulation of mitophagy in response to mitochondrial depolarization	OPTN
positive regulation of mitochondrial membrane potential	AKT2
mitochondrial membrane organization	ATP5F1A/ATP5PO/ALKBH7
mitochondrial DNA replication	RRM2B
positive regulation of autophagy of mitochondrion in response to mitochondrial depolarization	OPTN
regulation of autophagy of mitochondrion in response to mitochondrial depolarization	OPTN
mitochondrial transcription	MRPL12
positive regulation of mitochondrial translation	UQCC2
mitochondrial transport	SAE1/ATP5F1A/ATP5PO/ALKBH7
response to mitochondrial depolarisation	OPTN
positive regulation of autophagy of mitochondrion	OPTN
mitochondrial cytochrome c oxidase assembly	COX17
mitochondrial transmembrane transport	ATP5F1A/ATP5PO
mitochondrial respiratory chain complex assembly	COX17/UQCC2
regulation of mitochondrial translation	UQCC2
regulation of mitochondrial gene expression	UQCC2
positive regulation of protein targeting to mitochondrion	SAE1
positive regulation of mitochondrion organization	SAE1/OPTN
regulation of autophagy of mitochondrion	OPTN
regulation of protein targeting to mitochondrion	SAE1
positive regulation of establishment of protein localization to mitochondrion	SAE1
regulation of mitochondrion organization	SAE1/OPTN
regulation of establishment of protein localization to mitochondrion	SAE1
regulation of mitochondrial membrane potential	AKT2
regulation of mitochondrial membrane permeability	ALKBH7
autophagy of mitochondrion	OPTN
mitochondrion disassembly	OPTN
protein targeting to mitochondrion	SAE1
establishment of protein localization to mitochondrion	SAE1
protein localization to mitochondrion	SAE1
